# Evaluating test–retest reliability and sex‐/age‐related effects on temporal clustering coefficient of dynamic functional brain networks

**DOI:** 10.1002/hbm.26202

**Published:** 2023-01-13

**Authors:** Yicheng Long, Xuan Ouyang, Chaogan Yan, Zhipeng Wu, Xiaojun Huang, Weidan Pu, Hengyi Cao, Zhening Liu, Lena Palaniyappan

**Affiliations:** ^1^ Department of Psychiatry, and National Clinical Research Center for Mental Disorders The Second Xiangya Hospital of Central South University Changsha Hunan China; ^2^ CAS Key Laboratory of Behavioral Science, Institute of Psychology Chinese Academy of Sciences Beijing China; ^3^ Department of Psychology University of Chinese Academy of Sciences Beijing China; ^4^ International Big‐Data Center for Depression Research, Institute of Psychology Chinese Academy of Sciences Beijing China; ^5^ Department of Psychiatry Jiangxi Provincial People's Hospital, The First Affiliated Hospital of Nanchang Medical College Nanchang China; ^6^ Medical Psychological Institute The Second Xiangya Hospital, Central South University Changsha China; ^7^ Center for Psychiatric Neuroscience Feinstein Institute for Medical Research Manhasset New York USA; ^8^ Division of Psychiatry Research Zucker Hillside Hospital Glen Oaks New York USA; ^9^ Department of Psychiatry University of Western Ontario London Ontario Canada; ^10^ Robarts Research Institute University of Western Ontario London Ontario Canada; ^11^ Lawson Health Research Institute London Ontario Canada

**Keywords:** connectome, dynamic brain network, dynamic functional connectivity, functional connectivity, test–retest reliability

## Abstract

The multilayer dynamic network model has been proposed as an effective method to understand the brain function. In particular, derived from the definition of clustering coefficient in static networks, the temporal clustering coefficient provides a direct measure of the topological stability of dynamic brain networks and shows potential in predicting altered brain functions. However, test–retest reliability and demographic‐related effects on this measure remain to be evaluated. Using a data set from the Human Connectome Project (157 male and 180 female healthy adults; 22–37 years old), the present study investigated: (1) the test–retest reliability of temporal clustering coefficient across four repeated resting‐state functional magnetic resonance imaging scans as measured by intraclass correlation coefficient (ICC); and (2) sex‐ and age‐related effects on temporal clustering coefficient. The results showed that (1) the temporal clustering coefficient had overall moderate test–retest reliability (ICC > 0.40 over a wide range of densities) at both global and subnetwork levels, (2) female subjects showed significantly higher temporal clustering coefficient than males at both global and subnetwork levels, particularly within the default‐mode and subcortical regions, and (3) temporal clustering coefficient of the subcortical subnetwork was positively correlated with age in young adults. The results of sex effects were robustly replicated in an independent REST‐meta‐MDD data set, while the results of age effects were not. Our findings suggest that the temporal clustering coefficient is a relatively reliable and reproducible approach for identifying individual differences in brain function, and provide evidence for demographically related effects on the human brain dynamic connectomes.

## Graphical abstract

This study investigated the test–retest reliability and demographic‐related effects on a newly introduced dynamic graph‐based metric called temporal clustering coefficient in human functional brain networks. The main findings included that (1) the temporal clustering coefficient had overall moderate test–retest reliability at both global and subnetwork levels; (2) female subjects showed significantly higher temporal clustering coefficient than males at both global and subnetwork levels, particularly within the default‐mode and subcortical regions; and (3) temporal clustering coefficient of the subcortical subnetwork was positively correlated with age in young adults.

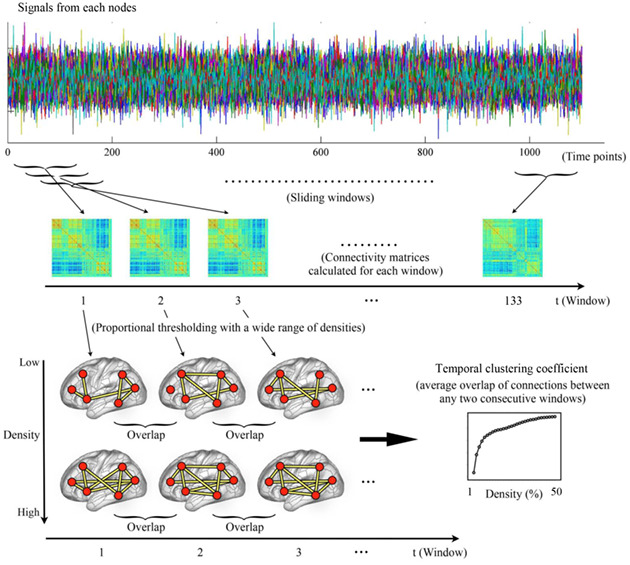

## 1 INTRODUCTION

Over the past decade, functional magnetic resonance imaging (fMRI)‐based brain graphs provide an effective way to model the Human Brain Connectome (Bullmore & Bassett, 2011; Cao et al., 2014). Several topological properties, such as the clustering coefficient and local efficiency that quantify the functional segregation and specialization of brain network (Rubinov & Sporns, 2010; Sporns, 2013), have been widely applied to both healthy and psychiatric populations (Liu et al., 2008; Suo et al., 2018; Tian et al., 2011; Zhang et al., 2011).

Conventional fMRI‐based brain graphs are constructed based on the assumption that functional connectivity (FC) patterns are stationary over the period of data acquisition. Recently, however, it has been demonstrated that FC in the brain fluctuates over time, implying that important information may be ignored when describing brain connectivity in a static manner (Chang & Glover, 2010; Hutchison, Womelsdorf, Gati, et al., 2013; Lurie et al., 2020). Thus, the investigation of dynamic FC (dFC) has rapidly developed which provides new insight into the brain's dynamical functions (D. Huang, Liu, et al., 2021; Hutchison, Womelsdorf, Allen, et al., 2013; Long, Liu, et al., 2020; Preti et al., 2017; Valsasina et al., 2019). A commonly used approach for this topic is to model the brain as a multilayer dynamic network (or “temporal network”) in a graph theory‐based framework, where a dynamic graph consists of multiple time‐ordered layers, and each layer corresponds to a “snapshot” of the brain's functional organization at a particular time point (Boccaletti et al., 2014; De Domenico, 2017; Pedersen et al., 2018). This is an explicit modeling framework that allows us to track where and when entities in a brain network transit from different subsystems, and has greatly enhanced our understanding of the human brain function (Pedersen & Zalesky, 2021).

In the model of dynamic graphs, spatio‐temporal properties of the brain network such as “temporal clustering” (Ding et al., 2020; Long, Cao, et al., 2020; Ren et al., 2017; Sizemore & Bassett, 2018; Zhao et al., 2021) and “temporal efficiency” (Dai et al., 2016; Fam et al., 2020; Long, Cao, et al., 2020; Sizemore & Bassett, 2018; Sun et al., 2019; Zhao et al., 2021) can be evaluated. These measures are derived from the definitions of analogous metrics for conventional static networks. Especially, the “temporal clustering coefficient,” which is also named “temporal correlation coefficient” by some researchers, quantifies the average topological overlap between any two consecutive layers (i.e., neighboring time points) of the connections in a dynamic brain network (Long, Cao, et al., 2020; Nicosia et al., 2013; Sizemore & Bassett, 2018; Tang et al., 2010). Compared with previous approaches quantifying standard deviations/average dissimilarities of dFC patterns across all layers (Long, Liu, et al., 2020; Marusak et al., 2017; Zhang et al., 2018; J. Zhang, Cheng, et al., 2016), the temporal clustering coefficient would thus serve as a more direct measure of a network's tendency to be stable over time. Some recent studies have suggested potential (patho)physiological correlates of this measure: for example, a significantly decreased temporal clustering coefficient of brain networks was found to be associated with greater cognitive workloads under complex task conditions in healthy subjects (Ren et al., 2017) and the presence of major depressive disorder during rest (Long, Cao, et al., 2020). These reports highlight the potential of the temporal clustering coefficient in predicting alterations in brain functions in both normal and pathological conditions.

To date, however, at least two empirical questions remain to be addressed before we can use the temporal clustering coefficient for large‐scale clinical studies. The first question relates to its test–retest reliability; a measure with limited test–retest stability would constrain its clinical utility in the study of individual differences and longitudinal studies (Z. Yang, Telesford, et al., 2021). While previous studies have investigated test–retest reliabilities of some measures of dFC stability such as the mean, variance, and standard deviation of dFC across different time windows (Choe et al., 2017; Zhang et al., 2018), relatively little is known if there are suitable topological metrics to reliably evaluate the dynamic features of the whole brain network. An exception is the “flexibility” (or called “switching rate”) based on dynamic community detection algorithms, which has been suggested to be a reliable measure of stability in modular structures of brain networks (Z. Yang, Telesford, et al., 2021). Nonetheless, the test–retest reliability of brain network flexibility was found to largely depend on the optimization of key parameters in the algorithms, which are data set‐specific and difficult to generalize (Z. Yang, Telesford, et al., 2021). Considering these limitations, an investigation of the reliability of the temporal clustering coefficient would be especially attractive since it allows direct inferences on the topological stability of brain networks (with no parameters to be set in advance) over time.

The second question refers to whether measures of the temporal clustering coefficient would be influenced by sex and age, two critical demographic variables in terms of clinical neuroscience research (Douw et al., 2011; Tian et al., 2011; C. Zhang, Cahill, et al., 2016). These two variables are known to be related to multiple brain network features and they are often incorporated into brain connectome analyses. For example, it has been found that the clustering coefficient is higher in females than in males (C. Zhang, Cahill, et al., 2016) and declines with the age (Masuda et al., 2018). These findings not only highlight the need to control for sex and age effects in neuroimaging studies, but also provide valuable insight into the sex/age differences in clinical characteristics of many diseases (e.g., females are more likely to present with depression than males [Eid et al., 2019]). Especially, several dFC‐based metrics including the earlier‐mentioned brain network flexibility have also been reported to be associated with sex and age (Mao et al., 2017; Tang et al., 2022; Xia et al., 2019). As a metric derived from brain dFC patterns, it might be hypothesized that the temporal clustering coefficient is also potentially affected by sex and/or age. However, this question is still unclear and needs to be ascertained to our knowledge.

In the present study, we aimed to address the above questions using a publicly available resting‐state fMRI (rs‐fMRI) data set from the Human Connectome Project (HCP) (Van Essen et al., 2013) to investigate: (1) test–retest reliability of temporal clustering coefficient and (2) possible sex‐ and age‐related effects on temporal clustering coefficient. To assess the stability of the findings, the derived results were further examined using different analytic pipelines (e.g., the window lengths) and validated in an independent data set. For comparative purposes, analogous metrics for conventional static brain networks (e.g., the clustering coefficient [Rubinov & Sporns, 2010]) were also calculated and compared to the findings in the dynamic networks.

## 2 MATERIALS AND METHODS

### 2.1 Data sets and data preprocessing

In line with a previous study (Ji et al., 2019), the analyzed sample consisted of 337 healthy subjects from the “S1200” release of the HCP data set (Van Essen et al., 2013) with no family relations. All participants were 22–37 years old with an average age of (28.61 ± 3.69) years; there were 157 males and 180 females. Each subject underwent four separate rs‐fMRI runs during 2 days, with a repetition time (TR) of 0.72 s and a length of 1200 time points for each run. The rs‐fMRI data of each run were preprocessed via HCP convention including the ICA‐FIX denoising steps, which was thought to be a more selective and effective approach to remove nonneural spatio‐temporal components of fMRI signals compared with conventional global signal regression (Glasser et al., 2013; Glasser et al., 2018; Glasser et al., 2019; Marcus et al., 2013). Further details about the sample information and preprocessing schemes can be found in the Supplemental Materials and previous publications (Glasser et al., 2013; Glasser et al., 2018; Ji et al., 2019; Marcus et al., 2013).

Informed consent was obtained from each participant as directed by the institutional review board at Washington University at St. Louis. This study was approved by the Ethics Committee of Second Xiangya Hospital, Central South University in Changsha, China.

### 2.2 Dynamic brain networks and temporal clustering coefficient

**FIGURE 1:**
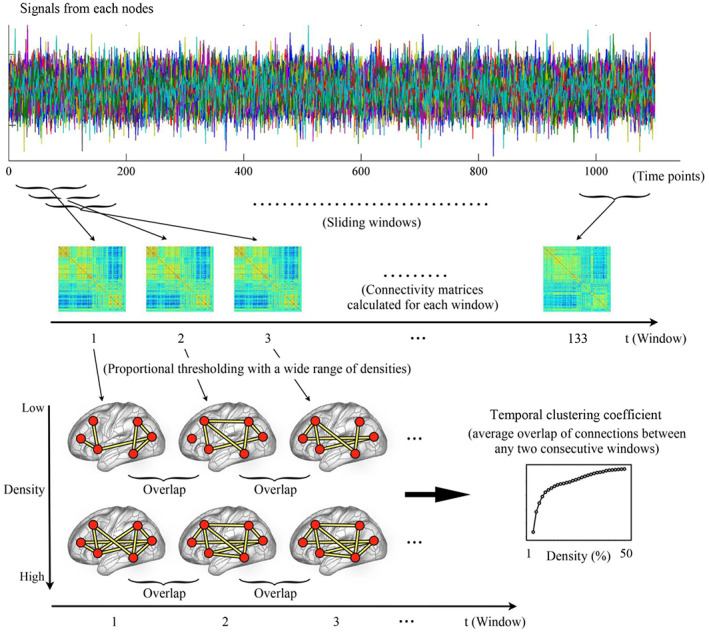
The steps for constructing dynamic brain networks and calculating temporal clustering coefficient, which include dividing the resting‐state functional magnetic resonance imaging (rs‐fMRI) signals into successive time windows; constructing binary brain networks within each time window with a wide range of density; and calculating the temporal clustering coefficients at each density by the overall probability for all connections in the network to persist over consecutive time windows (refer to Section 2 for details)

A dynamic network is comprised of a set of nodes and connections between nodes, in which the connections change over time (Holme & Saramäki, 2012; Sizemore & Bassett, 2018). Here, we defined the nodes in brain networks by two different parcellation atlases: (1) the automated anatomical labeling (AAL) atlas (Tzourio‐Mazoyer et al., 2002) which subdivides the brain into 90 regions of interest (ROIs) and (2) the Power functional atlas (Power et al., 2011) with 264 ROIs. Both these two atlases have been proved to be valid (Cao et al., 2014) and widely applied in various fMRI studies (Cao et al., 2018; Cao et al., 2021; Long et al., 2019; Tan et al., 2020). All following analyses were repeatedly performed using the two parcellation schemes separately. For each rs‐fMRI run of each subject, a multilayer dynamic brain network was then constructed by the sliding‐window approach (Laumann et al., 2017; Long, Cao, et al., 2020; Reinen et al., 2018) and the temporal clustering coefficient was computed, which was summarized below (also see Figure 1):

1. *Sliding‐windows*: First, the mean rs‐fMRI signals were extracted from each of the 90 or 264 nodes, which were then divided into a series of successive and partly overlapping time windows. Here, a fixed window width of 139 TRs (100.08 s) and a window sliding step length of 8 TRs (5.76 s) were used in the primary analyses, dividing the whole scan into a total of 133 windows. Note that selections of such parameters were based on recommendations in previous works to balance the quality of connectivity estimation and computational complexity (Long, Cao, et al., 2020; Sun et al., 2019; Zalesky & Breakspear, 2015), and the effects of changing the window width or sliding step length were also investigated in the latter part of this study. Within each window, the dFC strengths between all pairs of nodes were computed using Pearson correlations, resulting in a 90 × 90 or 264 × 264 connectivity matrix.
2. *Proportional thresholding*: The temporal clustering coefficient is currently defined in binary networks (Sizemore & Bassett, 2018), which are often obtained by thresholding the networks to preserve only the strongest connections between nodes (Bullmore & Sporns, 2009). In human brain functional connectomics, this was typically achieved by applying a strategy of proportional thresholding (Achard & Bullmore, 2007; Song et al., 2014; van den Heuvel et al., 2017). Here, we applied proportional thresholding on the dFC matrices of each window by preserving only a particular proportion, which is generally called the “density” (Bullmore & Sporns, 2009; Jalili, 2016), of the strongest connections between all possible pairs of nodes. The thresholding was applied with a wide range of densities ranging from 1 to 50% with an increment interval of 1% to ensure that results would not be biased by a single threshold (Bilbao et al., 2018; Cao, McEwen, et al., 2019). After thresholding, all the preserved connections were assigned a value of 1, and those not surviving the threshold were assigned a value of 0. As a result, a multilayer dynamic network *G* = (*G*_*t*_)_*t* = 1, 2, 3, …, 133_, where *G*_*t*_ is a binary network representing the “snapshot” of dFC pattern within the *t*th time window, was acquired at each density level.
3. *Temporal clustering coefficient*: At each density, the temporal clustering coefficient was calculated to quantify the overall probability for all connections in the network to persist over consecutive time points. The *nodal temporal clustering coefficient* was first computed for each node, as defined by the average overlapping of its connected neighbors between any two consecutive time windows. Briefly, let a_*ij*_ (*t*) = 1 if node *i* and node *j* are connected (being neighbors) within the *t*th time window, and a_*ij*_ (*t*) = 0 if they are not. The nodal temporal clustering coefficient of node *i* (*C*_*i*_) was then computed as
$$C_{i}=\frac{1}{T-1}\sum_{t=1}^{T-1}\frac{\sum_{j}a_{\mathrm{ij}}\left(t\right)a_{\mathrm{ij}}\left(t+1\right)}{\sqrt{\left[\sum_{j}a_{\mathrm{ij}}\left(t\right)\right]\left[\sum_{j}a_{\mathrm{ij}}\left(t+1\right)\right]}},$$

where [$\sum_{j}a_{\mathrm{ij}}\left(t\right)]$ equals to the number of nodes connected to node *i* within the *t*th time window; $[\sum_{j}a_{\mathrm{ij}}\left(t+1\right)$] equals to the number of nodes connected to node *i* within the (*t* + 1)th time window; [$\sum_{j}a_{\mathrm{ij}}\left(t\right)a_{\mathrm{ij}}\left(t+1\right)$] equals to the number of nodes which are consistently connected to node *i* within both the *t*th and (*t* + 1)th time windows; and *T* is the total number of time windows and equals to 133 here (Sizemore & Bassett, 2018; Tang et al., 2010). A higher *C*_*i*_ indicates that node *i* is more consistently connected with other nodes over time on average. After that, the global temporal clustering coefficient was obtained by averaging the nodal temporal clustering coefficients of all the 90 or 264 nodes (Sizemore & Bassett, 2018). Both the nodal and global temporal clustering coefficients range from 0 to 1, and higher values indicate higher tendencies for the connections to persist across multiple time windows.

4 *Subnetwork‐level temporal clustering coefficients*: According to previously published work (Cao, Chung, et al., 2019; Long et al., 2019; Mohr et al., 2016; Power et al., 2011), ROIs in both the two parcellation schemes can be assigned into nine subsystems including the default‐mode, salience, visual, subcortical, auditory, frontoparietal, cinguloopercular, sensorimotor, and attention subnetworks. Therefore, we further computed the temporal clustering coefficients for each of these individual subsystems, by averaging the nodal temporal clustering coefficients of all ROIs belonging to that subnetwork. Details about ROI assignments of each subnetwork can be found in Supplemental Tables 1 and 2.

### 2.3 Static network metrics for comparison

For exploratory comparative purposes, two analogous metrics in conventional static networks, the clustering coefficient and local efficiency (Rubinov & Sporns, 2010) were computed in the binary static brain networks constructed by the whole rs‐fMRI scans of each run. Here, they were computed at all densities ranging from 1 to 50%, and at both the global and subnetwork (by averaging the nodal clustering coefficients or nodal local efficiencies of all nodes belonging to particular subnetworks) levels, too.

### 2.4 Test–retest reliability

As in previous studies (Braun et al., 2012; Cao et al., 2014; Du et al., 2015; Jing et al., 2018; Wu et al., 2022; Z. Yang, Telesford, et al., 2021), the test–retest reliability of temporal clustering coefficient was evaluated by the intraclass correlation coefficient (ICC) utilizing a two‐way mixed, single measure model (ICC(3,1)) (Shrout & Fleiss, 1979) with time (multiple fMRI scans) as a fixed effect and subjects as a random effect. Specifically, it was calculated as

$$\mathrm{ICC}\left(3,1\right)=\frac{\mathrm{BMS}-\mathrm{EMS}}{\mathrm{BMS}+\left(k-1\right)*\mathrm{EMS}}$$

where *BMS* is the between‐subjects mean square (between‐subjects variance of the measures), *EMS* is the within‐subject error mean square and *k* is the number of repeated rs‐fMRI scans (equals to 4 here). We calculated ICC(3,1) here since we are interested in the consistency of measures between scans as it reflects, and it is commonly used in studies on test–retest reliabilities of fMRI measures over time (Elliott et al., 2020; Han et al., 2022; Noble et al., 2021). The ICCs were separately calculated at all densities, and at both the global and subnetwork levels. For comparative purposes, ICCs of the two static network metrics (i.e., the clustering coefficient and local efficiency) were also calculated.

The overall ICCs of all metrics were further obtained by averaging across all densities and reported descriptively (Braun et al., 2012; Deuker et al., 2009). According to overall ICCs, the reliabilities of each metrics were classified into four levels using the following criterion: poor (ICC < 0.4), moderate (0.4 ≤ ICC < 0.6), good (0.6 ≤ ICC < 0.75), and excellent (ICC > 0.75) (Cicchetti, 1994; Roach et al., 2019; Wu et al., 2022; Xiang et al., 2021). This criterion has been widely used in prior fMRI studies (Braun et al., 2012; Cao et al., 2014; Compère et al., 2021; Deuker et al., 2009; Gesierich et al., 2020), although more stringent criteria (e.g., considering ICCs under 0.5 to be poor) were also recommended (Jia et al., 2020; Nettekoven et al., 2018).

### 2.5 Sex‐ and age‐related effects

The possible sex‐ and age‐related effects in temporal clustering coefficients were investigated as follows: (1) for each subject, the temporal clustering coefficient at each density was obtained by averaging all the four rs‐fMRI runs; (2) the temporal clustering coefficients were then compared between the male and female subjects using a repeated‐measures analysis of covariance (ANCOVA) model in which density (from 1 to 50%) was included as within‐subject factor and sex as between‐subject factor, covarying for age, years of education and head motion (as measured by the mean framewise displacement (Power et al., 2012) across all the four runs); (3) the associations between age and temporal clustering coefficients were investigated by partial Spearman correlations adjusted for sex, education, and head motion, where temporal clustering coefficients were averaged over all densities before the correlation analyses. Note here we used nonparametric Spearman correlation rather than Pearson's correlation due to the non‐normal distribution of age in the current sample. Moreover, such model also allows to test both linear and nonlinear age effects on the temporal clustering coefficients; for the same reason, we chose to assess age and sex effects separately rather than in the same ANCOVA models, as performed in prior work (A. S. Huang, Rogers, et al., 2021). Furthermore, previous fMRI studies have highlighted that education level may account for part of the relationship between brain functions and age (Archer et al., 2018); therefore, even though there is no significant difference in years of education between the male and female participants (*t* = −0.351, *p* = .726), education was added as a covariate in all analyses to minimize possible confounding effects. All comparisons and correlation analyses were performed at both the global and subnetwork levels. The results were Bonferroni corrected for multiple tests (e.g., 2 tests at the global level, and 2 × 9 = 18 tests at the subnetwork level) and considered significant when corrected *p* < .05. Potential sex‐by‐age interaction effects on the temporal clustering coefficients (at both global and subnetwork levels) have been examined before investigating the sex and age effects, and no significant interactions were found (corrected‐*p* > .05). For comparative purposes, sex‐ and age‐related effects in the clustering coefficient and local efficiency were also investigated by the same methods.

### 2.6 Effects of analyzing strategies

To confirm whether the results would be affected by different analyzing strategies such as different window widths used when constructing dynamic brain networks, several additional analyses were performed. First, we reran the analyses on the temporal clustering coefficient with a wide range of different window widths (56/83/111/208 TRs, ≈40/60/80/150 s) used when keeping the sliding step length unchanged (8 TRs). Second, we reran the analyses on the temporal clustering coefficient with a wide range of different sliding step lengths (6/14/28 TRs, ≈4/10/20 s) used when keeping the window width unchanged (139 TRs).

### 2.7 Validation analysis

To assess the stability of the findings of sex‐ and age‐related effects on the temporal clustering coefficient, we further replicated the analyses in an independent validation data set with a close sex ratio and range of age. The validation data set consists of 529 young healthy adults (242 males and 287 females; 21–39 years old with an average age of 26.41 ± 5.26 years), which was drawn from the publicly available REST‐meta‐MDD data set (http://rfmri.org/REST-meta-MDD) (Yan et al., 2019). In the REST‐meta‐MDD project, all data have been deidentified and anonymized. Local Institutional Review Boards have approved all contributing studies and written informed consents were signed by all participants. More details can be found in the Supplemental Materials and previous publications using the REST‐meta‐MDD data set (Long, Cao, et al., 2020; Tang et al., 2022; Yan et al., 2019; H. Yang, Chen, et al., 2021).

### 2.8 Supplementary analyses

While we focused on the global and subnetwork‐level temporal clustering coefficients in this study, it would also be meaningful to know if the temporal clustering coefficients are reliable at the node‐wise level, and if such reliabilities are homogeneous across nodes in different areas. As supplementary analyses, we thus additionally calculated the ICCs of nodal temporal clustering coefficients for each node using the same methods as in the main analyses (averaging across the densities from 1 to 50%). Similarly, ICCs of the nodal clustering coefficients or nodal local efficiencies were also calculated for comparative purposes.

## 3 RESULTS

### 3.1 Test–retest reliabilities

**FIGURE 2:**
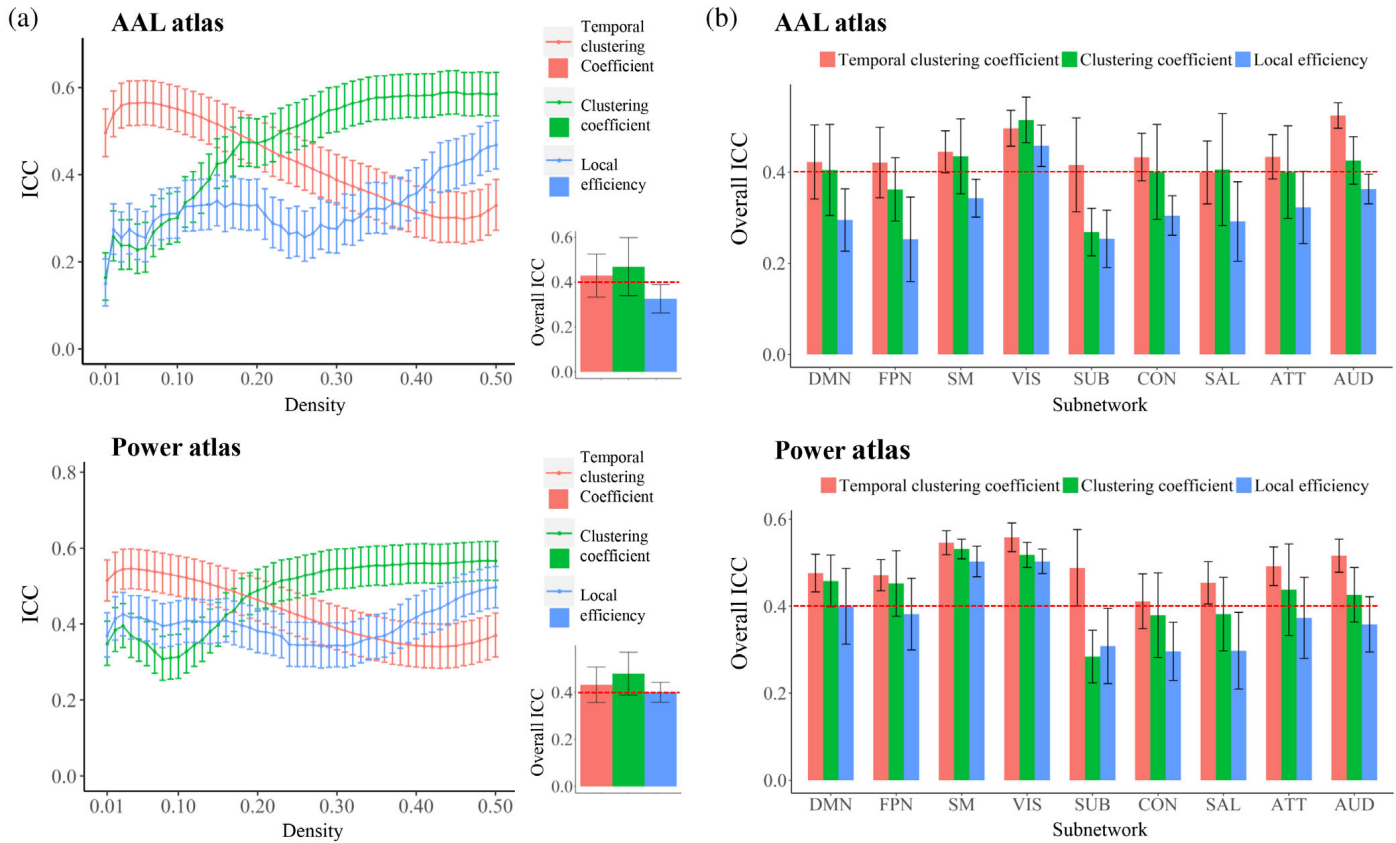
(a) Mean values of the intraclass correlation coefficient (ICC) at each density, and the overall ICCs obtained by averaging across all densities (the lower right corner) for each metric at the global level. (b) The overall ICCs for each metric at the subnetwork level. the error bars indicate 95% confidence intervals for ICCs or standard deviations for overall ICCs. ATT, attention subnetwork; AUD, auditory subnetwork; CON, cinguloopercular subnetwork; DMN, default‐mode subnetwork; FPN, frontoparietal subnetwork; SAL, salience subnetwork; SM, sensorimotor subnetwork; SUB, subcortical subnetwork; VIS, visual subnetwork

As shown in Figure 2a and Supplemental Table 3, the ICC values of all metrics varied across different densities: the ICC of temporal clustering coefficient varied in the ranges of 0.298–0.565 and 0.340–0.545; the ICC of clustering coefficient varied in the ranges of 0.164–0.589 and 0.308–0.567; and the ICC of local efficiency varied in the ranges of 0.150–0.468 and 0.342–0.497, when using the AAL and Power atlases, respectively.

When averaged across all densities, the overall ICC values of all metrics were mostly in the range of 0.40–0.59, which suggests a moderate test–retest reliability: the temporal clustering coefficient showed an overall ICC of 0.429 ± 0.096 (SD)/0.432 ± 0.075 (SD) when using the AAL/Power atlas; the clustering coefficient showed an average ICC of 0.469 ± 0.129 (SD)/0.480 ± 0.092 (SD) when using the AAL/Power atlas; the local efficiency showed an average ICC of 0.326 ± 0.063 (SD)/0.400 ± 0.043 (SD) when using the AAL/Power atlas (Figure 2a).

At the subnetwork level, as shown in Figure 2b and Supplemental Table 4, overall ICCs of the temporal clustering coefficients were found to be higher than 0.4 for most subnetworks which suggests a moderate test–retest reliability. Meanwhile, overall ICCs of the clustering coefficient and local efficiency for most subnetworks were found to be relatively lower than those of the temporal clustering coefficients for the same subnetworks (Figure 2b and Supplemental Table 4).

### 3.2 Sex‐ and age‐related effects

**FIGURE 3:**
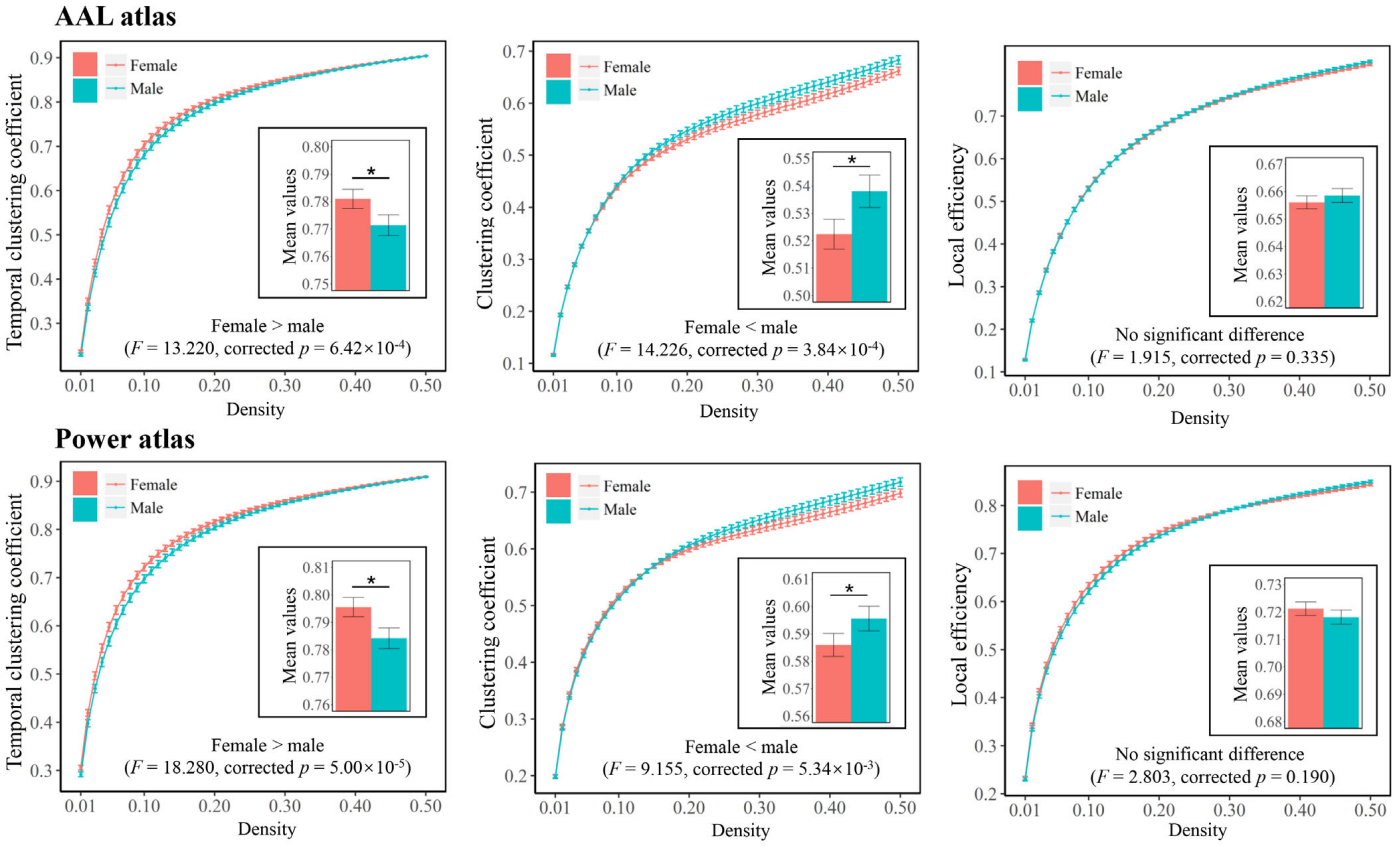
Comparisons of each metric at the global level between the male and female subjects. The mean values for each metric obtained by averaging across all densities (0.01–0.50) are also presented. Bonferroni‐corrected *p* values are presented. The “*” indicates a significant difference and error bars indicate 95% confidence intervals

**FIGURE 4:**
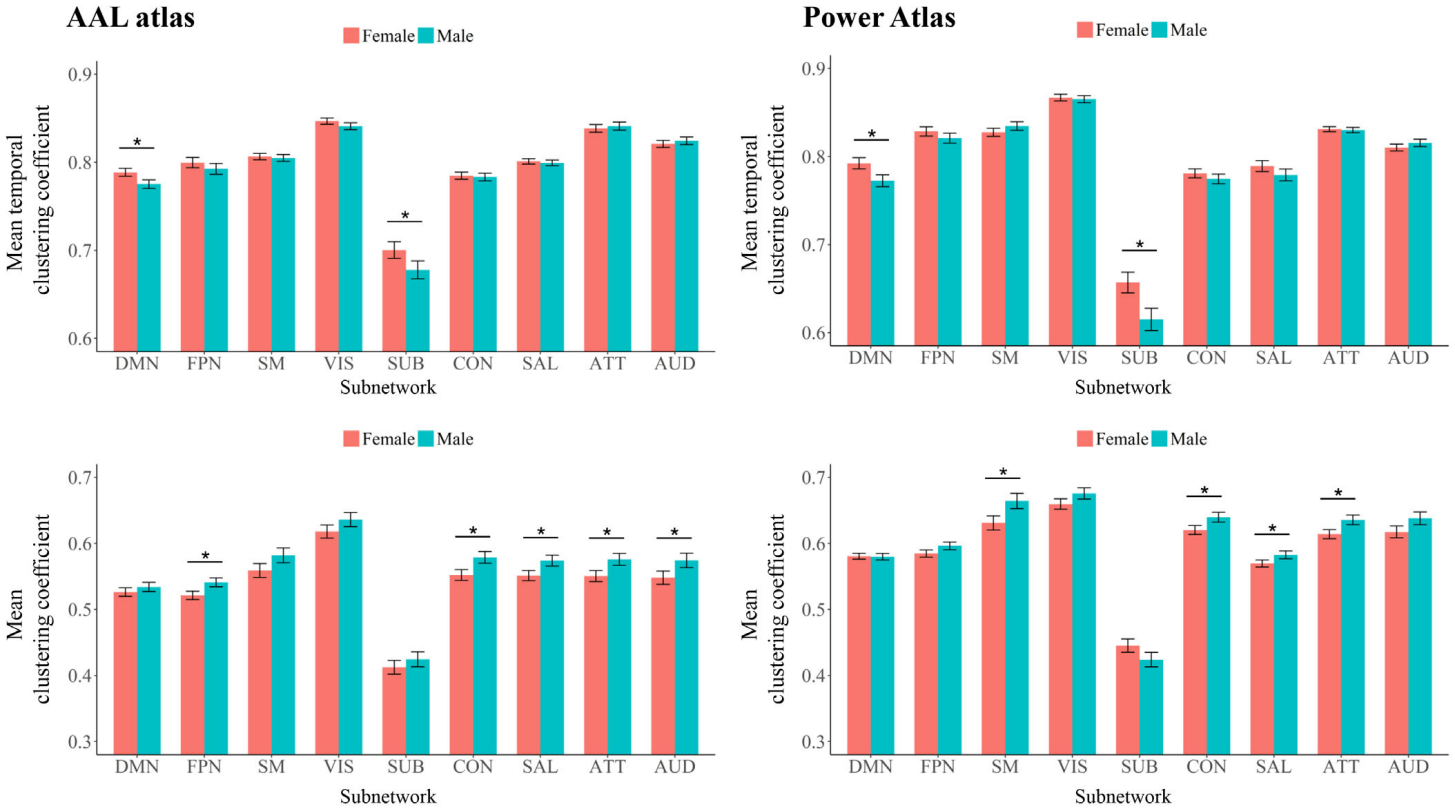
Comparisons of subnetwork‐level temporal clustering coefficients and clustering coefficients between the male and female subjects. The mean values for each metric obtained by averaging across all densities (0.01–0.50) are presented here. The “*” indicates a significant difference (with Bonferroni‐corrected *p* < .05) and error bars indicate 95% confidence intervals. ATT, attention subnetwork; AUD, auditory subnetwork; CON, cinguloopercular subnetwork; DMN, default‐mode subnetwork; FPN, frontoparietal subnetwork; SAL, salience subnetwork; SM, sensorimotor subnetwork; SUB, subcortical subnetwork; VIS, visual subnetwork

As shown in Figures 3 and 4 and Supplemental Table 5, female subjects showed a significantly higher global temporal clustering coefficient than male subjects (*F* = 13.220/18.280, Bonferroni‐corrected *p* = 6.42 × 10^−4^/5.00 × 10^−5^ when using the AAL/Power atlases). At the subnetwork level, significantly high temporal clustering coefficients in female subjects were found within the default‐mode and subcortical subnetworks (Bonferroni‐corrected *p* < .05), while no significant sex differences were found in other subnetworks (corrected *p* > .05). All the above results were robustly found with both the two parcellation schemes (AAL/Power atlases). As for static network metrics, a significantly lower clustering coefficient was found in females at both global (*F* = 14.226/9.115, Bonferroni‐corrected *p* = 3.84 × 10^−4^/5.34 × 10^−3^ when using the AAL/Power atlases) and subnetwork (Bonferroni‐corrected *p* < .05 for multiple subnetworks) levels, while no significant sex effects were found on the local efficiency (corrected *p* > .05).

**FIGURE 5:**
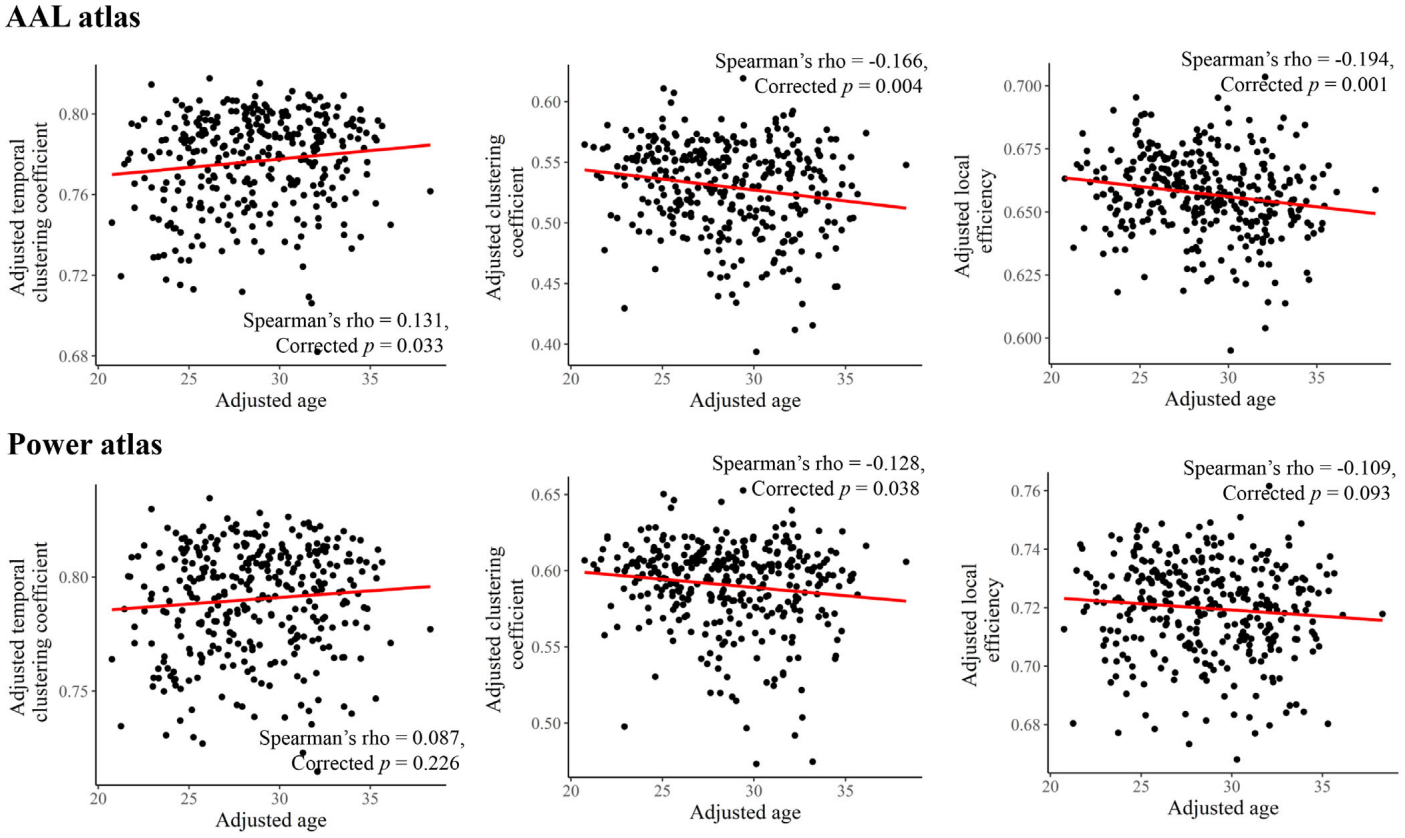
Results of partial Spearman correlations between age and each brain network metric at global level, after adjusting for sex, education, and head motion (Bonferroni‐corrected *p* values are presented). All metrics were averaged across all densities (0.01–0.50) before the correlation analyses

**FIGURE 6:**
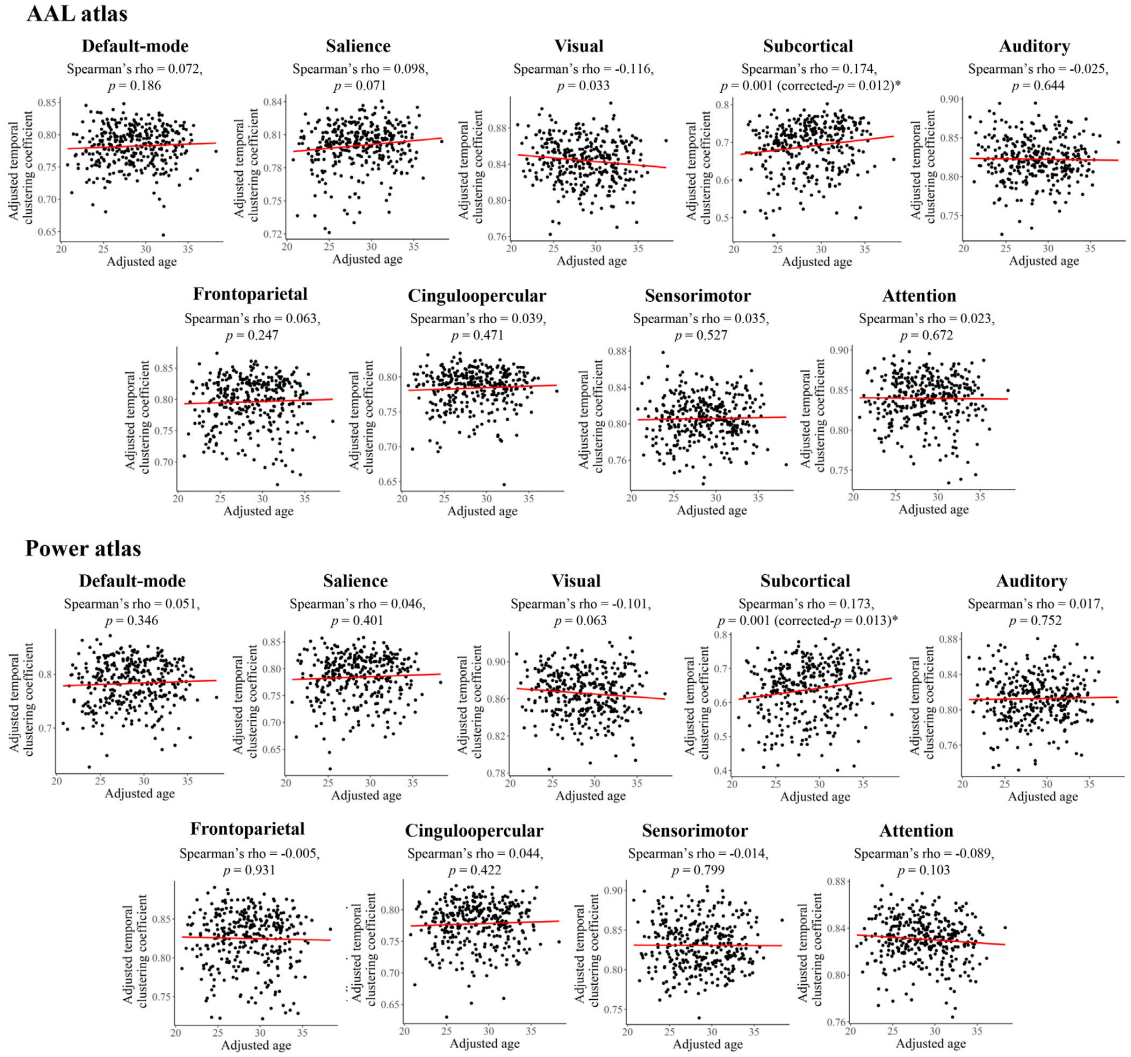
Results of partial Spearman correlations between age and temporal clustering coefficients of each subnetwork, after adjusting for sex, education, and head motion. The temporal clustering coefficients were averaged across all densities (0.01–0.50) before the correlation analyses. Uncorrected *p* values are presented and the “*” indicates a significant correlation with Bonferroni‐corrected *p* < .05

Results of the age effects are presented in Figures 5 and 6. At the global level, the results showed a positive relationship between age and temporal clustering coefficient which was, however, not significant based on the Power parcellation (Spearman's rho = 0.131/0.087, Bonferroni‐corrected *p* = .033/.226 when using the AAL/Power atlases). At the subnetwork level, a significant positive correlation was found between age and temporal clustering coefficient of the subcortical subnetwork (Spearman's rho = 0.174/0.173, uncorrected *p* = .001/.001, and Bonferroni corrected *p* = .012/.013 when using the AAL/Power atlases) with both the two parcellation schemes. As for static network metrics, at the global level, significant negative correlations were found between age and clustering coefficient when using both the AAL and Power atlases, as well as between age and local efficiency when using the AAL atlas (Bonferroni‐corrected *p* < .05); however, no significant correlations were found at the subnetwork level (corrected *p* > .05 for all subnetworks).

### 3.3 Effects of window widths/sliding step lengths

As shown in Tables 1 and 2, when using a wide range of different window widths and sliding step lengths, the temporal clustering coefficient consistently showed an overall ICC that is higher than 0.4, which suggests a fair test–retest reliability. The female subjects consistently showed a significantly higher global temporal clustering coefficient than male subjects (Bonferroni‐corrected *p* < .05, Tables 1 and 2); moreover, a significant positive correlation between age and temporal clustering coefficient of the subcortical subnetwork was consistently found when using different window widths and step lengths (corrected *p* < .05, Supplemental Tables 6 and 7).

**TABLE 1:** The test–retest reliabilities and sex differences of the global temporal clustering coefficients calculated with different window widths (the sliding step length was set at eight TRs here)

Window width	Overall ICC (mean ± standard deviation)		Significance of sex differences (female > male)	
	AAL atlas	Power atlas	AAL atlas	Power atlas
56 TRs	0.467 ± 0.084	0.495 ± 0.051	*F* = 22.271, corrected *p* = 7.00 × 10^−6^	*F* = 31.925, corrected *p* = 6.90 × 10^−8^
83 TRs	0.440 ± 0.094	0.461 ± 0.064	*F* = 17.139, corrected *p* = 8.82 × 10^−5^	*F* = 24.711, corrected *p* = 2.14 × 10^−6^
111 TRs	0.434 ± 0.095	0.442 ± 0.071	*F* = 14.784, corrected *p* = 2.88 × 10^−4^	*F* = 20.821, corrected *p* = 1.42 × 10^−5^
139 TRs	0.429 ± 0.096	0.432 ± 0.075	*F* = 13.220, corrected *p* = 6.42 × 10^−4^	*F* = 18.280, corrected *p* = 5.00 × 10^−5^
208 TRs	0.424 ± 0.095	0.419 ± 0.083	*F* = 11.633, corrected *p* = 1.46 × 10^−3^	*F* = 15.767, corrected *p* = 1.76 × 10^−4^

**TABLE 2:** The test–retest reliabilities and sex differences of the global temporal clustering coefficients calculated with different sliding step lengths (the window width was set at 139 TRs here)

Sliding step length	Overall ICC (mean ± standard deviation)		Significance of sex differences (female > male)	
	AAL	Power	AAL	Power
6 TRs	0.427 ± 0.100	0.425 ± 0.082	*F* = 12.750, corrected *p* = 8.18 × 10^−4^	*F* = 17.629, corrected *p* = 6.90 × 10^−4^
8 TRs	0.429 ± 0.096	0.432 ± 0.075	*F* = 13.220, corrected *p* = 6.42 × 10^−4^	*F* = 18.280, corrected *p* = 5.00 × 10^−5^
14 TRs	0.433 ± 0.084	0.448 ± 0.060	*F* = 14.690, corrected *p* = 3.04 × 10^−4^	*F* = 19.991, corrected *p* = 2.14 × 10^−5^
28 TRs	0.442 ± 0.060	0.477 ± 0.036	*F* = 17.834, corrected *p* = 6.24 × 10^−5^	*F* = 22.133, corrected *p* = 7.48 × 10^−6^

### 3.4 Validation analysis

In the validation data set, findings of sex effects on the temporal clustering coefficient were almost completely replicated, including both a significantly higher temporal clustering coefficient at the global level, as well as significantly higher temporal clustering coefficients within the default‐mode and subcortical subnetworks in females than males (Bonferroni‐corrected *p* < .05, see Supplemental Figure 1). However, age effects on the temporal clustering coefficients were not replicated in the validation data set (corrected *p* > .05).

### 3.5 Supplementary analyses

**FIGURE 7:**
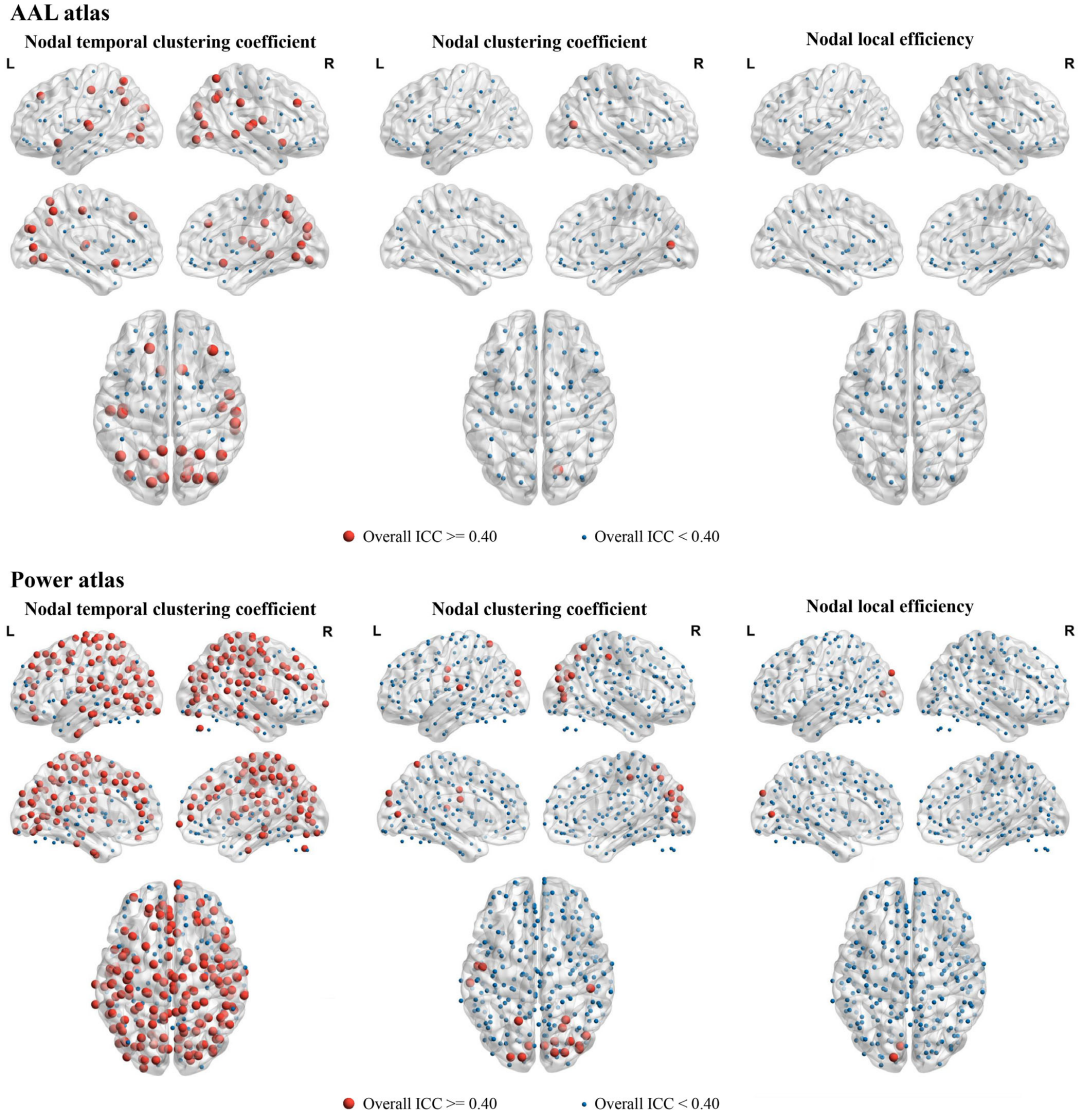
The spatial maps of intraclass correlation coefficients (ICCs) for each nodal‐level metric, where the nodes with an overall ICC >0.4 and an overall ICC <0.4 were marked with different colors

The spatial maps of overall ICCs for each node‐wise metric were shown in Figure 7. The spatial maps revealed findings consistent with those of subnetwork‐level metrics. First, for most nodes, the ICCs of nodal temporal clustering coefficients were relatively higher than those of examined static network metrics (nodal clustering coefficients and nodal local efficiencies); second, the ICCs of all nodal metrics were generally higher in cortical areas than subcortical areas, and higher when using the Power atlas than AAL atlas. Nevertheless, ICCs of the nodal temporal clustering coefficients were found to be lower when compared to the global and subnetwork‐level measures (over ICCs >0.4 for only part of the nodes). Therefore, we propose that it may be better and more reliable to estimate the temporal clustering coefficient at the global and large‐scale subnetwork levels, compared with the level of single nodes.

## 4 DISCUSSION

In this study, we investigated test–retest reliability and sex‐ and age‐related effects on a newly introduced dynamic graph‐based metric called temporal clustering coefficient in human functional brain networks. Overall, our results support three main conclusions. First, the temporal clustering coefficient showed moderate test–retest reliability at both global and subnetwork levels, which is comparable to those of analogous conventional static brain network metrics (e.g., clustering coefficient and local efficiency). Second, significantly higher temporal clustering coefficients in female subjects than in males were found at the global level and within the default‐mode and subcortical regions. Third, a significant positive relationship was found between age and temporal clustering coefficient at the subnetwork level within the subcortical regions.

The first major contribution of this study is the evaluation of test–retest reliability of the temporal clustering coefficient in human brain network for the first time to our knowledge. The temporal clustering coefficient (also named temporal correlation coefficient) has previously been used to characterize the persistence of connections over time in time‐varying systems such as the public transportation system (Galati et al., 2013), trade network (Büttner et al., 2016), and the human brain functional networks (Long, Cao, et al., 2020; Ren et al., 2017; Sizemore & Bassett, 2018). However, it is unknown about the reliability of this approach when applied to human brains. In this study, our results revealed fair test–retest reliability (0.4 < overall ICC < 0.5) for temporal clustering coefficients in both the whole brain and individual large‐scale subnetworks (Figure 2), based on a common guideline where ICCs between 0.4 and 0.6 are considered moderate (Braun et al., 2012; Cao et al., 2014; Compère et al., 2021; Deuker et al., 2009; Gesierich et al., 2020). However, it is notable that ICCs under 0.5 would be considered “poor” when using some more stringent cutoffs (Jia et al., 2020; Nettekoven et al., 2018). Hence, we consider the temporal clustering coefficient to be a relatively reliable metric to characterize the brain functional dynamics as supported by the results.

The second important finding of our study is a sex difference in the temporal clustering coefficient. Using two different parcellation schemes, a significantly higher temporal clustering coefficient was consistently observed in female subjects than in males, for both the entire brain (Figure 3) and the default‐mode and subcortical systems at the subnetwork level (Figure 4). These results were also replicated in an independent validation data set (Supplemental Figure 1). A higher “temporal clustering” indicates a higher average overlap of the network structures between consecutive time points (Sizemore & Bassett, 2018). Our results thus, intriguingly, suggest a higher trend for dFC patterns to be persistent over time in the brains of females than those of males. To our knowledge, investigations on differences in dFC between males and females are still limited, with various methods employed and inconsistent results (de Lacy et al., 2019; Mao et al., 2017; Menon & Krishnamurthy, 2019; Yaesoubi et al., 2015). For instance, by comparing the “flexibility” as defined by switching frequency of the network modular community structures, an earlier study has reported higher flexibility in females than males within regions of the default‐mode network, thus suggesting lower temporal stability of dFC patterns in females (Mao et al., 2017). Another research explored the brain dynamics by using a state‐clustering algorithm and suggested that females switch connectivity states less frequently and spend more time in the state where dFC within the default‐mode network is strong (de Lacy et al., 2019). Here, our results were in line with the latter one. Considering that our results were obtained from a relatively large sample and confirmed with different parcellation schemes/different data sets, the present study may provide further evidence that female subjects have temporally more stable functional brain organizations than males, especially within the default‐mode and subcortical regions. Notably, alterations in temporal stability of dFC within these systems have been associated with some common mental diseases such as major depressive disorder (Long, Cao, et al., 2020; Wise et al., 2017) and bipolar disorder (Nguyen et al., 2017), which have marked sex biases in incidences (Seedat et al., 2009). Therefore, it is hypothesized that altered dFC may play important roles in such sex differences, which can be further investigated in future studies.

Besides the sex differences, our results also showed a significant positive correlation between age and the temporal clustering coefficient of the subcortical subnetwork (Figure 6). There were some previous studies based on different methodologies reporting that temporal variability of dFC “states” involving the subcortical regions was negatively correlated with age (Xia et al., 2019). Therefore, our results were partly in line with the previous report, and may suggest increasing temporal stability (decreasing temporal variability) of the subcortical dFC patterns during the process of aging. However, apart from the subcortical regions, significant age effects on dFC variability within some other brain systems such as the default‐mode cortices have also been reported (Park et al., 2017; Qin et al., 2015) but they were not observed in this study. One possible reason for such instability of results is that the age range of the current sample is relatively narrow (22–37 years old). In future studies, it might be necessary to investigate if similar age effects on the temporal clustering coefficient would exist over a broader range of age or even lifespan. Moreover, it is noteworthy that we failed to replicate the age effects on the temporal clustering coefficient in an independent validation data set; therefore, such findings should be treated with caution and may need to be further confirmed in other samples. Nevertheless, our results may provide initial evidence for the sex and age effects on the temporal clustering coefficient, indicating that these two variables should be taken into consideration when interpreting the results of the temporal clustering coefficient in further studies.

In recent years, there have been some efforts to understand how the brain's functional organization fluctuates over time, pointing out that it is temporally changed in specific manners (Ma & Zhang, 2018; Vidaurre et al., 2017). It has been suggested that, for instance, the dFC patterns of the brain do not fluctuate randomly but tend to persist over some periods (Allen et al., 2014; Tang et al., 2010). Based on this important property, the activity of human brains during rest can be temporally clustered into discrete short periods or “connectivity states,” during which the dFC patterns remain quasi‐stable over tens of seconds (Damaraju et al., 2014; Leonardi et al., 2013). Furthermore, alterations related to such connectivity states have been reported in various psychiatric disorders (Damaraju et al., 2014; Rashid et al., 2014; Reinen et al., 2018; Zhi et al., 2018). Despite these accumulating findings, little has been known as to whether there is a reliable measure to quantify the tendency for dFC patterns to persist over time, and to detect the interindividual differences in such tendencies. Here, we propose that the measure of the temporal clustering coefficient may be a suitable indicator of temporal persistence of the brain's functional structures, and could potentially be used to predict alterations in brain functions given its acceptable reliability and high reproducibility as shown by our results.

For exploratory comparative purposes, we repeated the whole analysis on two analogous static network metrics including the clustering coefficient and local efficiency. It was found that the temporal clustering coefficient had a close overall ICC compared with these two metrics at the global level (Figure 2a). Moreover, the temporal clustering coefficient had relatively higher ICCs than those of the clustering coefficient and local efficiency for most subnetworks at the local level; and especially, the difference is most pronounced for the subcortical subnetwork (Figure 2b). As a possible explanation, we consider this is because subcortical structures such as the thalamus and basal ganglia relay and modulate information passing to different areas of the brain; their dFC patterns are most variable over time since they may connect with different brain regions and be involved in different functional communities/modules at different times (Long, Liu, et al., 2020; J. Zhang, Cheng, et al., 2016). Therefore, it might be more precise to characterize these regions by dFC rather than conventional static FC. As for sex‐ and age‐related effects, several significant results were obtained for both the clustering coefficient and local efficiency, which are in line with some previous research (Figures 3, 4, 5) (Douw et al., 2011; Tian et al., 2011). Importantly, it was shown that compared with the clustering coefficient and local efficiency, the temporal clustering coefficient showed significant sex‐/age‐related effects within different subnetworks, indicating that they may reflect different aspects of brain function. This may partly support the opinion that dFC can capture important information ignored by static methodology (Chang & Glover, 2010; Hutchison, Womelsdorf, Gati, et al., 2013), and further highlight the value of temporal clustering coefficient.

We noted that reliability of the temporal clustering coefficient decreased with density level (ICCs are highest at densities around 0.01–0.10, and ICCs <0.4 when density is higher than 0.30) (Figure 2). We speculate that it is because when more links are added to the network, the nodes in the network become more uniformly connected, thereby reducing potential network variability (Braun et al., 2012); therefore, it might be more sensitive and specific to use low‐density graphs to capture the temporal variability of dynamic network structures. Interestingly, such a trend is opposite to the patterns of most conventional static brain network metrics (whose reliabilities are usually poor when density <0.10 but increased with density level) (Braun et al., 2012). Therefore, we propose that in future studies, it may be suitable to use a density range of 0.10–0.30 when simultaneously evaluating the temporal clustering coefficient and static network metrics.

To date, there remain debates concerning the optimal window width and step length of the sliding windows in the constructions of dynamic brain networks (Qin et al., 2015; Savva et al., 2020; Zhang et al., 2018). Nevertheless, it was found that the main conclusions of this study were robust to changes in the window widths and step lengths (Tables 1 and 2). As mentioned above, most results were also largely unaffected by using different parcellation schemes when defining nodes in the brain networks. Therefore, our results are unlikely to be mainly driven by these factors.

There are some limitations of this study and possible future directions to be noted. First, the temporal clustering coefficient is currently defined based on binary graphs derived by thresholding the correlations (Sizemore & Bassett, 2018). However, the process of thresholding may throw away some important information such as “weak” but neurobiologically meaningful connections, and information contained in the connection weights (Cao et al., 2014; Rubinov & Sporns, 2010). The definition of temporal clustering coefficient may be expanded to weighted graphs in future studies to deal with such weakness. Second, in both the HCP and REST‐meta‐MDD data sets, the participants were identified as “male” or “female” based on a binary scale, and we are unable to examine whether biological sex and gender identity may have differentially influence on the brain connectome (Hyatt et al., 2022). Based on the current data, we are also unable to control for some potential confounding factors such as parity, oral contraceptive, and hormonal replacement therapy status which may regulate the brain structures and functions (de Lange et al., 2020; Taylor et al., 2021; Voldsbekk et al., 2021). Third, the age range of the current sample is relatively narrow and our results need to be confirmed in participants with a broader age range. Fourth, while we performed all analyses using two validated atlases for node definition and a commonly used rs‐fMRI experiment, it may be necessary to confirm our results with some other parcellation schemes and tasks. Fifth, the four rs‐fMRI runs in the HCP data set were scanned with different phase‐encoding directions (i.e., left‐to‐right and right‐to‐left) which might lead to different distortion effects; although the distortions have been carefully corrected using the HCP preprocessing pipeline (Glasser et al., 2013), it is still possible that the distortion‐related effects were not fully mitigated and could influence dFCs as well as their reliabilities, which merits further investigations in future studies. Finally, exploring possible alterations in the temporal clustering coefficient in psychiatric populations, rather than only in healthy participants, may provide much more important clinical implications for brain functional dynamics.

## 5 CONCLUSIONS

In summary, in this study, we investigated the test–retest reliability of a novel dynamic graph‐based metric called temporal clustering coefficient, and possible sex‐ and age‐related effects on this measure in young healthy adults. The results demonstrated fair reliability at both global and subnetwork levels, suggesting that it could be a relatively reliable approach to identify individual differences in brain connectome. Moreover, a significantly higher temporal clustering coefficient was found in females than males both at the global level and at the local level within the default‐mode and subcortical subnetworks, suggesting a temporally more stable functional brain organization in females. At the local level, a significant positive correlation was found between age and the temporal clustering coefficient of the subcortical subnetwork, which may suggest increased temporal stability of subcortical dFC patterns along with age. However, the age effects should be treated with caution since it was not replicated in an independent data set. These results may serve as an initial guide for evaluating human brain functional dynamics using the temporal clustering coefficient.

## AUTHOR CONTRIBUTIONS

**Yicheng Long**: Conceptualization, data curation, formal analysis, funding acquisition, methodology, visualization, writing ‐ original draft. **Xuan Ouyang**: Resources, funding acquisition, writing ‐ review & editing. **Chaogan Yan**: Supervision, writing ‐ review & editing. **Zhipeng Wu**: Writing ‐ review & editing. **Xiaojun Huang**: Funding acquisition, writing ‐ review & editing. **Weidan Pu**: Funding acquisition, writing ‐ review & editing. **Hengyi Cao**: Conceptualization, formal analysis, methodology, writing ‐ review & editing. **Zhening Liu**: Resources, data curation, funding acquisition, supervision, writing ‐ review & editing. **Lena Palaniyappan**: Conceptualization, supervision, writing ‐ review & editing.

## CONFLICT OF INTEREST

The authors report no competing conflict of interests.

## Supporting information


**APPENDIX S1** Supporting Information

## Data Availability

The “S1200” release of the HCP data set is publicly available on the HCP website (https://www.humanconnectome.org/study/hcp-young-adult). The REST‐meta‐MDD data set is publicly available at: http://rfmri.org/REST-meta-MDD. The temporal clustering coefficient was computed based on a publicly available MATLAB toolbox (Sizemore & Bassett, 2018); the codes can be found at: https://github.com/Yicheng-Long/dynamic_graph_metrics. The clustering coefficient and local efficiency were computed using the Brain Connectivity Toolbox (http://www.brain-connectivity-toolbox.net) (Rubinov & Sporns, 2010).
