# Supplementary material for: Evaluating test–retest reliability and sex‐/age‐related effects on temporal clustering coefficient of dynamic functional brain networks
Source: Hum Brain Mapp. 2023 Jan 13;44(6):2191–208. doi: 10.1002/hbm.26202 (PMC10028647; doi:10.1002/hbm.26202)
Supplement: Supplementary file 1 — APPENDIX S1 Supporting Information [file HBM-44-2191-s001.docx]

**Evaluating test-retest reliability and sex/age-related effects on temporal clustering coefficient of dynamic functional brain networks**

***Supplemental Information***

**SUPPLEMENTAL METHODS**

**Datasets and data preprocessing**

Data in the present study was drawn from the “S1200” release of the Human Connectome Project (HCP) dataset (Van Essen et al., 2013). In line with a previous study (Ji et al., 2019), we selected all subjects with no family relations, resulting in a total of 337 subjects included in the analyses. Four resting-state functional magnetic resonance imaging (rs-fMRI) sessions were collected for each participant using the Gradient-echo EPI with the following data acquisition parameters: repetition time = 720 ms, echo time = 33.1 ms, flip angle = 52°, filed of view = 208 × 180 mm, matrix = 104 × 90, slice thickness = 2.0 mm, 72 slices, 2.0 mm isotropic voxels, and time points = 1200 (scanning time = 14.5 mins) for each session. We downloaded the FIX-Denoised data which was preprocessed with the conventional HCP functional and ICA-FIX pipelines (Glasser et al., 2018, 2013; Marcus et al., 2013). More details about the sample information and preprocessing schemes can be found in the reference manual of the HCP data release at: <https://www.humanconnectome.org/storage/app/media/documentation/s1200/HCP_S1200_Release_Reference_Manual.pdf>.

**Dynamic Brain Networks and Temporal Clustering Coefficient**

Nodes in brain networks were defined by two different parcellation atlases: 1) the Automated Anatomical Labeling (AAL) atlas (Tzourio-Mazoyer et al., 2002) with 90 regions of interest (ROIs) and 2) the Power functional atlas (Power et al., 2011) with 264 ROIs. Referring to previously published work (Cao et al., 2019; Long et al., 2019; Mohr et al., 2016; Power et al., 2011), ROIs in both the two parcellation schemes were assigned into 9 subsystems including the default-mode, salience, visual, subcortical, auditory, frontoparietal, cinguloopercular, sensorimotor and attention subnetworks. Details about ROI assignments of each subnetwork can be found in **Supplemental Tables 1-2**.

**Validation analysis**

The validation dataset was drawn from the REST-meta-MDD Project, which is a multi-site publicly-available rs-fMRI dataset (http://rfmri.org/REST-meta-MDD) (Yan et al., 2019). All healthy subjects in the REST-meta-MDD Project who met the following criteria were included: 1) 21~39 years of age; 2) demographic information such as sex is complete; 3) fMRI scanning repetition time = 2 s (to minimize biases caused by different temporal resolutions when constructing dynamic brain networks); 4) image quality and spatial normalization are satisfactory determined by manual checking; 5) mean framewise-displacement (FD) < 0.2 mm; 6) good mask coverage with no signal loss in any ROI.

Data of the REST-meta-MDD project was preprocessed using a standardized pipeline including removing the first 10 volumes, slice timing, motion realignment, brain tissue segmentation, spatial normalization, temporal filtering (0.01-0.10Hz), and nuisance regression. More details of the pipeline can be found in earlier publications (Long et al., 2020; Tang et al., 2022; Yan et al., 2019; Yang et al., 2021).

After data preprocessing, dynamic brain network construction and temporal clustering coefficient calculation were performed in the same way as we did in the primary analysis (the window width was set as 100 seconds and the window sliding step length was set as 6 seconds). Sex- and age-related effects on temporal clustering coefficient were also investigated in the same way as we did in the primary analysis, except that the site was included as an additional covariant (as dummy variables) in all statistical analyses.

**SUPPLEMENTAL RESULTS**

**Test-retest Reliabilities**

ICCs of all metrics at the global level (temporal clustering coefficient, clustering coefficient, and local efficiency) at each density were presented in **Supplemental Table 3**. Overall ICCs (averaged across the density from 1% to 50%) of all metrics at the subnetwork level were presented in **Supplemental Table 4**.

**Sex- and Age-related Effects**

A significantly higher global temporal clustering coefficient was found in female subjects than male subjects; comparisons at each density level were shown in **Supplemental Table 5**.

**Effects of Window Widths/Sliding Step Lengths**

When using different window widths and step length, a significant positive correlation between age and temporal clustering coefficient of the subcortical subnetwork was consistently found (corrected *p* < 0.05, **Supplemental** **Tables 6-7**).

**References**:

Cao, H., Chung, Y., McEwen, S.C., Bearden, C.E., Addington, J., Goodyear, B., Cadenhead, K.S., Mirzakhanian, H., Cornblatt, B.A., Carrión, R., Mathalon, D.H., McGlashan, T.H., Perkins, D.O., Belger, A., Seidman, L.J., Thermenos, H., Tsuang, M.T., van Erp, T.G.M., Walker, E.F., Hamann, S., Anticevic, A., Woods, S.W., Cannon, T.D., 2019. Progressive reconfiguration of resting-state brain networks as psychosis develops: Preliminary results from the North American Prodrome Longitudinal Study (NAPLS) consortium. Schizophr. Res. https://doi.org/10.1016/j.schres.2019.01.017

Glasser, M.F., Coalson, T.S., Bijsterbosch, J.D., Harrison, S.J., Harms, M.P., Anticevic, A., Van Essen, D.C., Smith, S.M., 2018. Using temporal ICA to selectively remove global noise while preserving global signal in functional MRI data. Neuroimage 181, 692–717. https://doi.org/10.1016/j.neuroimage.2018.04.076

Glasser, M.F., Sotiropoulos, S.N., Wilson, J.A., Coalson, T.S., Fischl, B., Andersson, J.L., Xu, J., Jbabdi, S., Webster, M., Polimeni, J.R., Van Essen, D.C., Jenkinson, M., 2013. The minimal preprocessing pipelines for the Human Connectome Project. Neuroimage 80, 105–124. https://doi.org/10.1016/j.neuroimage.2013.04.127

Ji, J.L., Spronk, M., Kulkarni, K., Repovš, G., Anticevic, A., Cole, M.W., 2019. Mapping the human brain’s cortical-subcortical functional network organization. Neuroimage 185, 35–57. https://doi.org/10.1016/j.neuroimage.2018.10.006

Long, Y., Cao, H., Yan, C., Chen, X., Li, L., Castellanos, F.X., Bai, T., Bo, Q., Chen, G., Chen, N., Chen, W., Cheng, C., Cheng, Y., Cui, X., Duan, J., Fang, Y., Gong, Q., Guo, W., Hou, Z., Hu, L., Kuang, L., Li, F., Li, K., Li, T., Liu, Y., Luo, Q., Meng, H., Peng, D., Qiu, H., Qiu, J., Shen, Y., Shi, Y., Si, T., Wang, C., Wang, F., Wang, K., Wang, L., Wang, X., Wang, Y., Wu, Xiaoping, Wu, Xinran, Xie, C., Xie, G., Xie, H., Xie, P., Xu, X., Yang, H., Yang, J., Yao, J., Yao, S., Yin, Y., Yuan, Y., Zhang, A., Zhang, H., Zhang, K., Zhang, L., Zhang, Z., Zhou, R., Zhou, Y., Zhu, J., Zou, C., Zang, Y., Zhao, J., Kin-yuen Chan, C., Pu, W., Liu, Z., 2020. Altered resting-state dynamic functional brain networks in major depressive disorder: Findings from the REST-meta-MDD consortium. NeuroImage Clin. https://doi.org/10.1016/j.nicl.2020.102163

Long, Y., Chen, C., Deng, M., Huang, X., Tan, W., Zhang, L., Fan, Z., Liu, Z., 2019. Psychological resilience negatively correlates with resting-state brain network flexibility in young healthy adults: a dynamic functional magnetic resonance imaging study. Ann. Transl. Med. 7, 809–809. https://doi.org/10.21037/atm.2019.12.45

Marcus, D.S., Harms, M.P., Snyder, A.Z., Jenkinson, M., Wilson, J.A., Glasser, M.F., Barch, D.M., Archie, K.A., Burgess, G.C., Ramaratnam, M., Hodge, M., Horton, W., Herrick, R., Olsen, T., McKay, M., House, M., Hileman, M., Reid, E., Harwell, J., Coalson, T., Schindler, J., Elam, J.S., Curtiss, S.W., Van Essen, D.C., 2013. Human Connectome Project informatics: Quality control, database services, and data visualization. Neuroimage 80, 202–219. https://doi.org/10.1016/j.neuroimage.2013.05.077

Mohr, H., Wolfensteller, U., Betzel, R.F., Mišić, B., Sporns, O., Richiardi, J., Ruge, H., 2016. Integration and segregation of large-scale brain networks during short-term task automatization. Nat. Commun. 7. https://doi.org/10.1038/ncomms13217

Power, J.D., Cohen, A.L., Nelson, S.M., Wig, G.S., Barnes, K.A., Church, J.A., Vogel, A.C., Laumann, T.O., Miezin, F.M., Schlaggar, B.L., Petersen, S.E., 2011. Functional Network Organization of the Human Brain. Neuron 72, 665–678. https://doi.org/10.1016/j.neuron.2011.09.006

Tang, S., Wu, Z., Cao, H., Chen, X., Wu, G., Tan, W., Liu, D., Yang, J., Long, Y., Liu, Z., 2022. Age-Related Decrease in Default-Mode Network Functional Connectivity Is Accelerated in Patients With Major Depressive Disorder. Front. Aging Neurosci. 13. https://doi.org/10.3389/fnagi.2021.809853

Tzourio-Mazoyer, N., Landeau, B., Papathanassiou, D., Crivello, F., Etard, O., Delcroix, N., Mazoyer, B., Joliot, M., 2002. Automated anatomical labeling of activations in SPM using a macroscopic anatomical parcellation of the MNI MRI single-subject brain. Neuroimage 15, 273–289. https://doi.org/10.1006/nimg.2001.0978

Van Essen, D.C., Smith, S.M., Barch, D.M., Behrens, T.E.J., Yacoub, E., Ugurbil, K., 2013. The WU-Minn Human Connectome Project: An overview. Neuroimage 80, 62–79. https://doi.org/10.1016/j.neuroimage.2013.05.041

Yan, C.G., Chen, X., Li, L., Castellanos, F.X., Bai, T.J., Bo, Q.J., Cao, J., Chen, G.M., Chen, N.X., Chen, W., Cheng, C., Cheng, Y.Q., Cui, X.L., Duan, J., Fang, Y.R., Gong, Q.Y., Guo, W.B., Hou, Z.H., Hu, L., Kuang, L., Li, F., Li, K.M., Li, T., Liu, Y.S., Liu, Z.N., Long, Y.C., Luo, Q.H., Meng, H.Q., Peng, D.H., Qiu, H.T., Qiu, J., Shen, Y.D., Shi, Y.S., Wang, C.Y., Wang, F., Wang, K., Wang, L., Wang, X., Wang, Y., Wu, X.P., Wu, X.R., Xie, C.M., Xie, G.R., Xie, H.Y., Xie, P., Xu, X.F., Yang, H., Yang, J., Yao, J.S., Yao, S.Q., Yin, Y.Y., Yuan, Y.G., Zhang, A.X., Zhang, H., Zhang, K.R., Zhang, L., Zhang, Z.J., Zhou, R.B., Zhou, Y.T., Zhu, J.J., Zou, C.J., Si, T.M., Zuo, X.N., Zhao, J.P., Zang, Y.F., 2019. Reduced default mode network functional connectivity in patients with recurrent major depressive disorder. Proc Natl Acad Sci U S A 116, 9078–9083. https://doi.org/10.1073/pnas.1900390116

Yang, H., Chen, X., Chen, Z.-B., Li, L., Li, X.-Y., Castellanos, F.X., Bai, T.-J., Bo, Q.-J., Cao, J., Chang, Z.-K., Chen, G.-M., Chen, N.-X., Chen, W., Cheng, C., Cheng, Y.-Q., Cui, X.-L., Duan, J., Fang, Y., Gong, Q.-Y., Guo, W.-B., Hou, Z.-H., Hu, L., Kuang, L., Li, F., Li, H.-X., Li, K.-M., Li, T., Liu, Y.-S., Liu, Z.-N., Long, Y.-C., Lu, B., Luo, Q.-H., Meng, H.-Q., Peng, D., Qiu, H.-T., Qiu, J., Shen, Y.-D., Shi, Y.-S., Si, T.-M., Tang, Y.-Q., Wang, C.-Y., Wang, F., Wang, K., Wang, L., Wang, X., Wang, Y., Wang, Y.-W., Wu, X.-P., Wu, X.-R., Xie, C.-M., Xie, G.-R., Xie, H.-Y., Xie, P., Xu, X.-F., Yang, J., Yao, J.-S., Yao, S.-Q., Yin, Y.-Y., Yuan, Y.-G., Zang, Y.-F., Zhang, A.-X., Zhang, H., Zhang, K.-R., Zhang, L., Zhang, Z.-J., Zhao, J.-P., Zhou, R., Zhou, Y.-T., Zhu, J.-J., Zhu, Z.-C., Zou, C.-J., Zuo, X.-N., Yan, C.-G., 2021. Disrupted intrinsic functional brain topology in patients with major depressive disorder. Mol. Psychiatry. https://doi.org/10.1038/s41380-021-01247-2

**Supplemental Figure 1** Results of sex effects on the temporal clustering coefficient in the validation dataset. (**A**) Comparisons of temporal clustering coefficient at the global level between the male and female subjects. (**B**) Comparisons of subnetwork-level temporal clustering coefficients between the male and female subjects. The presented mean values of temporal clustering coefficients were obtained by averaging across all densities (0.01 to 0.50). The “*” indicates a significant difference (with Bonferroni-corrected *p* < 0.05) and error bars indicate 95% confidence intervals. ATT, attention subnetwork; AUD, auditory subnetwork; CON, cinguloopercular subnetwork; DMN, default-mode subnetwork; FPN, frontoparietal subnetwork; SAL, salience subnetwork; SM, sensorimotor subnetwork; SUB, subcortical subnetwork; VIS, visual subnetwork.


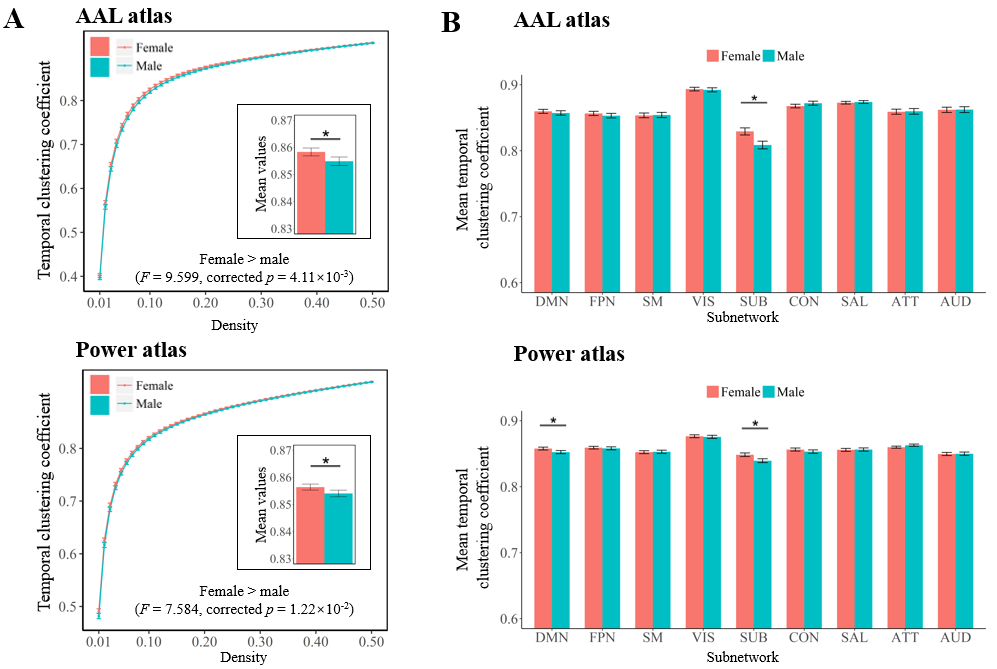


**Supplemental Table 1.** List of the 90 ROIs defined by the AAL atlas and their subnetwork assignments. Odd and even numbers stand for left and right hemispheres, respectively.

| Index | Labels | Subnetwork | Index | Labels | Subnetwork |
| --- | --- | --- | --- | --- | --- |
| (1,2) | Precentral gyrus | Sensorimotor | (47,48) | Lingual gyrus | Visual |
| (3,4) | Superior frontal gyrus, dorsolateral | Frontoparietal | (49,50) | Superior occipital gyrus | Visual |
| (5,6) | Superior frontal gyrus, orbital part | Frontoparietal | (51,52) | Middle occipital gyrus | Visual |
| (7,8) | Middle frontal gyrus | Salience/ frontoparietal/attention | (53,54) | Inferior occipital gyrus | Visual |
| (9, 10) | Middle frontal gyrus, orbital part | Frontoparietal | (55,56) | Fusiform gyrus | Visual |
| (11,12) | Inferior frontal gyrus, opercular part | Cingulo-opercular | (57,58) | Postcentral gyrus | Sensorimotor |
| (13,14) | Inferior frontal gyrus, triangular part | Salience/ frontoparietal/attention | (59,60) | Superior parietal gyrus | Salience/attention |
| (15,16) | Inferior frontal gyrus, orbital part | None | (61,62) | Inferior parietal, but supramarginal and angular gyri | Frontoparietal/attention |
| (17,18) | Rolandic operculum | Auditory/ cingulo-opercular | (63,64) | Supramarginal gyrus | Auditory/ cingulo-opercular |
| (19,20) | Supplementary motor area | Sensorimotor | (65,66) | Angular gyrus | Default-mode |
| (21,22) | Olfactory cortex | None | (67,68) | Precuneus | Default-mode |
| (23,24) | Superior frontal gyrus, medial | Default-mode | (69,70) | Paracentral lobule | Sensorimotor |
| (25,26) | Superior frontal gyrus, medial orbital | Default-mode | (71,72) | Caudate nucleus | Subcortical |
| (27,28) | Gyrus rectus | None | (73,74) | Lenticular nucleus, putamen | Subcortical |
| (29,30) | Insula | Salience/ cingulo-opercular | (75,76) | Lenticular nucleus, pallidum | Subcortical |
| (31,32) | Anterior cingulate and paracingulate gyri | Default-mode/ salience | (77,78) | Thalamus | Subcortical |
| (33,34) | Median cingulate and paracingulate gyri | Salience/ cingulo-opercular | (79,80) | Heschl gyrus | Auditory |
| (35,36) | Posterior cingulate gyrus | Default-mode | (81,82) | Superior temporal gyrus | Auditory/attention |
| (37,38) | Hippocampus | None | (83,84) | Temporal pole: superior temporal gyrus | Cingulo-opercular |
| (39,40) | Parahippocampal gyrus | Default-mode | (85,86) | Middle temporal gyrus | Default-mode |
| (41,42) | Amygdala | None | (87,88) | Temporal pole: middle temporal gyrus | Default-mode |
| (43,44) | Calcarine fissure and surrounding cortex | Visual | (89,90) | Inferior temporal gyrus | None |
| (45,46) | Cuneus | Visual |  |  |  |

**Supplemental Table 2.** List of the 264 ROIS defined by the Power atlas and their subnetwork assignments.

| Index | MNI (x) | MNI (y) | MNI (z) | Subnetwork |
| --- | --- | --- | --- | --- |
| 1 | -24 | -99 | -12 | None |
| 2 | 27 | -96 | -12 | None |
| 3 | 24 | 33 | -18 | None |
| 4 | -57 | -45 | -24 | None |
| 5 | 9 | 42 | -24 | None |
| 6 | -21 | -21 | -21 | None |
| 7 | 18 | -27 | -18 | None |
| 8 | -36 | -30 | -27 | None |
| 9 | 66 | -24 | -18 | None |
| 10 | 51 | -33 | -27 | None |
| 11 | 54 | -30 | -18 | None |
| 12 | 33 | 39 | -12 | None |
| 13 | -6 | -51 | 60 | Sensorimotor |
| 14 | -15 | -18 | 39 | Sensorimotor |
| 15 | 0 | -15 | 48 | Sensorimotor |
| 16 | 9 | -3 | 45 | Sensorimotor |
| 17 | -6 | -21 | 66 | Sensorimotor |
| 18 | -6 | -33 | 72 | Sensorimotor |
| 19 | 12 | -33 | 75 | Sensorimotor |
| 20 | -54 | -24 | 42 | Sensorimotor |
| 21 | 30 | -18 | 72 | Sensorimotor |
| 22 | 9 | -45 | 72 | Sensorimotor |
| 23 | -24 | -30 | 72 | Sensorimotor |
| 24 | -39 | -18 | 54 | Sensorimotor |
| 25 | 30 | -39 | 60 | Sensorimotor |
| 26 | 51 | -21 | 42 | Sensorimotor |
| 27 | -39 | -27 | 69 | Sensorimotor |
| 28 | 21 | -30 | 60 | Sensorimotor |
| 29 | 45 | -9 | 57 | Sensorimotor |
| 30 | -30 | -42 | 60 | Sensorimotor |
| 31 | 9 | -18 | 75 | Sensorimotor |
| 32 | 21 | -42 | 69 | Sensorimotor |
| 33 | -45 | -33 | 48 | Sensorimotor |
| 34 | -21 | -30 | 60 | Sensorimotor |
| 35 | -12 | -18 | 75 | Sensorimotor |
| 36 | 42 | -21 | 54 | Sensorimotor |
| 37 | -39 | -15 | 69 | Sensorimotor |
| 38 | -15 | -45 | 72 | Sensorimotor |
| 39 | 3 | -27 | 60 | Sensorimotor |
| 40 | 3 | -18 | 57 | Sensorimotor |
| 41 | 39 | -18 | 45 | Sensorimotor |
| 42 | -48 | -12 | 36 | Sensorimotor |
| 43 | 36 | -9 | 15 | Sensorimotor |
| 44 | 51 | -6 | 33 | Sensorimotor |
| 45 | -54 | -9 | 24 | Sensorimotor |
| 46 | 66 | -9 | 24 | Sensorimotor |
| 47 | -3 | 3 | 54 | Cingulo-opercular |
| 48 | 54 | -27 | 33 | Cingulo-opercular |
| 49 | 18 | -9 | 63 | Cingulo-opercular |
| 50 | -15 | -6 | 72 | Cingulo-opercular |
| 51 | -9 | -3 | 42 | Cingulo-opercular |
| 52 | 36 | 0 | -3 | Cingulo-opercular |
| 53 | 12 | 0 | 69 | Cingulo-opercular |
| 54 | 6 | 9 | 51 | Cingulo-opercular |
| 55 | -45 | 0 | 9 | Cingulo-opercular |
| 56 | 48 | 9 | 0 | Cingulo-opercular |
| 57 | -33 | 3 | 3 | Cingulo-opercular |
| 58 | -51 | 9 | -3 | Cingulo-opercular |
| 59 | -6 | 18 | 33 | Cingulo-opercular |
| 60 | 36 | 9 | 0 | Cingulo-opercular |
| 61 | 33 | -27 | 12 | Auditory |
| 62 | 66 | -33 | 21 | Auditory |
| 63 | 57 | -15 | 6 | Auditory |
| 64 | -39 | -33 | 18 | Auditory |
| 65 | -60 | -24 | 15 | Auditory |
| 66 | -48 | -27 | 6 | Auditory |
| 67 | 42 | -24 | 21 | Auditory |
| 68 | -51 | -33 | 27 | Auditory |
| 69 | -54 | -21 | 24 | Auditory |
| 70 | -54 | -9 | 12 | Auditory |
| 71 | 57 | -6 | 12 | Auditory |
| 72 | 60 | -18 | 30 | Auditory |
| 73 | -30 | -27 | 12 | Auditory |
| 74 | -42 | -75 | 27 | Default-mode |
| 75 | 6 | 66 | -3 | Default-mode |
| 76 | 9 | 48 | -15 | Default-mode |
| 77 | -12 | -39 | 0 | Default-mode |
| 78 | -18 | 63 | -19 | Default-mode |
| 79 | -45 | -60 | 21 | Default-mode |
| 80 | 42 | -72 | 27 | Default-mode |
| 81 | -45 | 12 | -33 | Default-mode |
| 82 | 45 | 15 | -30 | Default-mode |
| 83 | -69 | -24 | -15 | Default-mode |
| 84 | -57 | -27 | -15 | None |
| 85 | 27 | 15 | -18 | None |
| 86 | -45 | -66 | 36 | Default-mode |
| 87 | -39 | -75 | 45 | Default-mode |
| 88 | -6 | -54 | 27 | Default-mode |
| 89 | 6 | -60 | 36 | Default-mode |
| 90 | -12 | -57 | 15 | Default-mode |
| 91 | -3 | -48 | 12 | Default-mode |
| 92 | 9 | -48 | 30 | Default-mode |
| 93 | 15 | -63 | 27 | Default-mode |
| 94 | -3 | -36 | 45 | Default-mode |
| 95 | 12 | -54 | 18 | Default-mode |
| 96 | 51 | -60 | 36 | Default-mode |
| 97 | 24 | 33 | 48 | Default-mode |
| 98 | -9 | 39 | 51 | Default-mode |
| 99 | -15 | 30 | 54 | Default-mode |
| 100 | -36 | 21 | 51 | Default-mode |
| 101 | 21 | 39 | 39 | Default-mode |
| 102 | 12 | 54 | 39 | Default-mode |
| 103 | -9 | 54 | 39 | Default-mode |
| 104 | -21 | 45 | 39 | Default-mode |
| 105 | 6 | 54 | 15 | Default-mode |
| 106 | 6 | 63 | 21 | Default-mode |
| 107 | -6 | 51 | 0 | Default-mode |
| 108 | 9 | 54 | 3 | Default-mode |
| 109 | -3 | 45 | -9 | Default-mode |
| 110 | 9 | 42 | -6 | Default-mode |
| 111 | -12 | 45 | 9 | Default-mode |
| 112 | -3 | 39 | 36 | Default-mode |
| 113 | -3 | 42 | 15 | Default-mode |
| 114 | -21 | 63 | 18 | Default-mode |
| 115 | -9 | 48 | 24 | Default-mode |
| 116 | 66 | -12 | -18 | Default-mode |
| 117 | -57 | -12 | -9 | Default-mode |
| 118 | -57 | -30 | -3 | Default-mode |
| 119 | 66 | -30 | -9 | Default-mode |
| 120 | -69 | -42 | -6 | Default-mode |
| 121 | 12 | 30 | 60 | Default-mode |
| 122 | 12 | 36 | 21 | Default-mode |
| 123 | 51 | -3 | -15 | Default-mode |
| 124 | -27 | -39 | -9 | Default-mode |
| 125 | 27 | -36 | -12 | Default-mode |
| 126 | -33 | -39 | -15 | Default-mode |
| 127 | 27 | -78 | -33 | Default-mode |
| 128 | 51 | 6 | -30 | Default-mode |
| 129 | -54 | 3 | -27 | Default-mode |
| 130 | 48 | -51 | 30 | Default-mode |
| 131 | -48 | -42 | 0 | Default-mode |
| 132 | -30 | 18 | -18 | None |
| 133 | -3 | -36 | 30 | None |
| 134 | -6 | -72 | 42 | None |
| 135 | 12 | -66 | 42 | None |
| 136 | 3 | -48 | 51 | None |
| 137 | -45 | 30 | -12 | Default-mode |
| 138 | -9 | 12 | 66 | Attention |
| 139 | 48 | 36 | -12 | Default-mode |
| 140 | 9 | -90 | -6 | None |
| 141 | 18 | -90 | -15 | None |
| 142 | -12 | -96 | -12 | None |
| 143 | 18 | -48 | -9 | Visual |
| 144 | 39 | -72 | 15 | Visual |
| 145 | 9 | -72 | 12 | Visual |
| 146 | -9 | -81 | 6 | Visual |
| 147 | -27 | -78 | 18 | Visual |
| 148 | 21 | -66 | 3 | Visual |
| 149 | -24 | -90 | 18 | Visual |
| 150 | 27 | -60 | -9 | Visual |
| 151 | -15 | -72 | -9 | Visual |
| 152 | -18 | -69 | 6 | Visual |
| 153 | 42 | -78 | -12 | Visual |
| 154 | -48 | -75 | -9 | Visual |
| 155 | -15 | -90 | 30 | Visual |
| 156 | 15 | -87 | 36 | Visual |
| 157 | 30 | -78 | 24 | Visual |
| 158 | 21 | -87 | -3 | Visual |
| 159 | 15 | -78 | 30 | Visual |
| 160 | -15 | -51 | 0 | Visual |
| 161 | 42 | -66 | -9 | Visual |
| 162 | 24 | -87 | 24 | Visual |
| 163 | 6 | -72 | 24 | Visual |
| 164 | -42 | -75 | 0 | Visual |
| 165 | 27 | -78 | -15 | Visual |
| 166 | -15 | -78 | 33 | Visual |
| 167 | -3 | -81 | 21 | Visual |
| 168 | -39 | -87 | -6 | Visual |
| 169 | 36 | -84 | 12 | Visual |
| 170 | 6 | -81 | 6 | Visual |
| 171 | -27 | -90 | 3 | Visual |
| 172 | -33 | -78 | -12 | Visual |
| 173 | 36 | -81 | 0 | Visual |
| 174 | -45 | 3 | 45 | Frontoparietal |
| 175 | 48 | 24 | 27 | Frontoparietal |
| 176 | -48 | 12 | 24 | Frontoparietal |
| 177 | -54 | -48 | 42 | Frontoparietal |
| 178 | -24 | 12 | 63 | Frontoparietal |
| 179 | 57 | -54 | -15 | Frontoparietal |
| 180 | 24 | 45 | -15 | Frontoparietal |
| 181 | 33 | 54 | -12 | Frontoparietal |
| 182 | -21 | 42 | -21 | None |
| 183 | -18 | -75 | -24 | None |
| 184 | 18 | -81 | -33 | None |
| 185 | 36 | -66 | -33 | None |
| 186 | 48 | 9 | 33 | Frontoparietal |
| 187 | -42 | 6 | 33 | Frontoparietal |
| 188 | -42 | 39 | 21 | Frontoparietal |
| 189 | 39 | 42 | 15 | Frontoparietal |
| 190 | 48 | -42 | 45 | Frontoparietal |
| 191 | -27 | -57 | 48 | Frontoparietal |
| 192 | 45 | -54 | 48 | Frontoparietal |
| 193 | 33 | 15 | 57 | Frontoparietal |
| 194 | 36 | -66 | 39 | Frontoparietal |
| 195 | -42 | -54 | 45 | Frontoparietal |
| 196 | 39 | 18 | 39 | Frontoparietal |
| 197 | -33 | 54 | 3 | Frontoparietal |
| 198 | -42 | 45 | -3 | Frontoparietal |
| 199 | 33 | -54 | 45 | Frontoparietal |
| 200 | 42 | 48 | -3 | Frontoparietal |
| 201 | -42 | 24 | 30 | Frontoparietal |
| 202 | -3 | 27 | 45 | Frontoparietal |
| 203 | 12 | -39 | 51 | Salience |
| 204 | 54 | -45 | 36 | Salience |
| 205 | 42 | 0 | 48 | Salience |
| 206 | 30 | 33 | 27 | Salience |
| 207 | 48 | 21 | 9 | Salience |
| 208 | -36 | 21 | 0 | Salience |
| 209 | 36 | 21 | 3 | Salience |
| 210 | 36 | 33 | -3 | Salience |
| 211 | 33 | 15 | -9 | Salience |
| 212 | -12 | 27 | 24 | Salience |
| 213 | 0 | 15 | 45 | Salience |
| 214 | -27 | 51 | 21 | Salience |
| 215 | 0 | 30 | 27 | Salience |
| 216 | 6 | 24 | 36 | Salience |
| 217 | 9 | 21 | 27 | Salience |
| 218 | 30 | 57 | 15 | Salience |
| 219 | 27 | 51 | 27 | Salience |
| 220 | -39 | 51 | 18 | Salience |
| 221 | 3 | -24 | 30 | None |
| 222 | 6 | -24 | 0 | Subcortical |
| 223 | -3 | -12 | 12 | Subcortical |
| 224 | -9 | -18 | 6 | Subcortical |
| 225 | 12 | -18 | 9 | Subcortical |
| 226 | -6 | -27 | -3 | Subcortical |
| 227 | -21 | 6 | -6 | Subcortical |
| 228 | -15 | 3 | 9 | Subcortical |
| 229 | 30 | -15 | 3 | Subcortical |
| 230 | 24 | 9 | 0 | Subcortical |
| 231 | 30 | 0 | 3 | Subcortical |
| 232 | -30 | -12 | 0 | Subcortical |
| 233 | 15 | 6 | 6 | Subcortical |
| 234 | 9 | -3 | 6 | Subcortical |
| 235 | 54 | -42 | 21 | Attention |
| 236 | -57 | -51 | 9 | Attention |
| 237 | -54 | -39 | 15 | Attention |
| 238 | 51 | -33 | 9 | Attention |
| 239 | 51 | -30 | -3 | Attention |
| 240 | 57 | -45 | 12 | Attention |
| 241 | 54 | 33 | 0 | Attention |
| 242 | -48 | 24 | 0 | Attention |
| 243 | -15 | -66 | -21 | None |
| 244 | -33 | -54 | -24 | None |
| 245 | 21 | -57 | -24 | None |
| 246 | 0 | -63 | -18 | None |
| 247 | 33 | -12 | -33 | None |
| 248 | -30 | -9 | -36 | None |
| 249 | 48 | -3 | -39 | None |
| 250 | -51 | -6 | -39 | None |
| 251 | 9 | -63 | 60 | Attention |
| 252 | -51 | -63 | 6 | Attention |
| 253 | -48 | -51 | -21 | None |
| 254 | 45 | -48 | -18 | None |
| 255 | 48 | -30 | 48 | Sensorimotor |
| 256 | 21 | -66 | 48 | Attention |
| 257 | 45 | -60 | 3 | Attention |
| 258 | 24 | -57 | 60 | Attention |
| 259 | -33 | -45 | 48 | Attention |
| 260 | -27 | -72 | 36 | Attention |
| 261 | -33 | 0 | 54 | Attention |
| 262 | -42 | -60 | -9 | Attention |
| 263 | -18 | -60 | 63 | Attention |
| 264 | 30 | -6 | 54 | Attention |

**Supplemental Table 3.** ICCs of all metrics (at the global level) at each density.

| Density (%) | AAL atlas | | | Power atlas | | |
| --- | --- | --- | --- | --- | --- | --- |
|  | ICC | | | ICC | | |
|  | Temporal clustering coefficient | Clustering coefficient | Local efficiency | Temporal clustering coefficient | Clustering coefficient | Local efficiency |
| 1 | 0.495983 | 0.163786 | 0.150258 | 0.515124 | 0.348331 | 0.370517 |
| 2 | 0.539658 | 0.257324 | 0.273393 | 0.536448 | 0.384561 | 0.414507 |
| 3 | 0.558733 | 0.237254 | 0.255159 | 0.544699 | 0.395318 | 0.425649 |
| 4 | 0.563959 | 0.237497 | 0.274325 | 0.54611 | 0.369097 | 0.417101 |
| 5 | 0.564392 | 0.227778 | 0.261058 | 0.544477 | 0.358936 | 0.417037 |
| 6 | 0.565301 | 0.23126 | 0.255903 | 0.541598 | 0.347072 | 0.41177 |
| 7 | 0.564015 | 0.2674 | 0.293343 | 0.538188 | 0.326956 | 0.404447 |
| 8 | 0.560261 | 0.285255 | 0.307271 | 0.534579 | 0.308487 | 0.395366 |
| 9 | 0.555462 | 0.296843 | 0.310195 | 0.530543 | 0.311555 | 0.398378 |
| 10 | 0.550791 | 0.30134 | 0.310574 | 0.526306 | 0.313493 | 0.403467 |
| 11 | 0.544397 | 0.335763 | 0.326931 | 0.522016 | 0.328262 | 0.408731 |
| 12 | 0.538681 | 0.347617 | 0.327642 | 0.517055 | 0.345306 | 0.408981 |
| 13 | 0.532033 | 0.368195 | 0.329966 | 0.511787 | 0.358732 | 0.406331 |
| 14 | 0.523473 | 0.38869 | 0.330577 | 0.50531 | 0.380924 | 0.404805 |
| 15 | 0.515908 | 0.424731 | 0.339202 | 0.498815 | 0.398122 | 0.407143 |
| 16 | 0.507024 | 0.42884 | 0.325838 | 0.492506 | 0.424441 | 0.409131 |
| 17 | 0.499488 | 0.452329 | 0.334185 | 0.485907 | 0.436661 | 0.402226 |
| 18 | 0.490014 | 0.474352 | 0.330748 | 0.478321 | 0.464066 | 0.396155 |
| 19 | 0.48161 | 0.473985 | 0.328754 | 0.470766 | 0.479788 | 0.390698 |
| 20 | 0.471874 | 0.472655 | 0.330373 | 0.463976 | 0.4892 | 0.381581 |
| 21 | 0.461731 | 0.477354 | 0.307991 | 0.455936 | 0.496549 | 0.378997 |
| 22 | 0.452127 | 0.484665 | 0.288137 | 0.447887 | 0.507379 | 0.374479 |
| 23 | 0.44359 | 0.497574 | 0.282537 | 0.439881 | 0.514975 | 0.367969 |
| 24 | 0.436457 | 0.505412 | 0.263891 | 0.432017 | 0.517742 | 0.346077 |
| 25 | 0.429077 | 0.51139 | 0.267917 | 0.424108 | 0.522431 | 0.34509 |
| 26 | 0.420827 | 0.519278 | 0.256849 | 0.417596 | 0.528815 | 0.344694 |
| 27 | 0.413126 | 0.528261 | 0.264731 | 0.409801 | 0.532316 | 0.345063 |
| 28 | 0.404888 | 0.537876 | 0.284554 | 0.403169 | 0.538539 | 0.341502 |
| 29 | 0.397371 | 0.547829 | 0.277051 | 0.395651 | 0.54284 | 0.343325 |
| 30 | 0.387831 | 0.55012 | 0.275921 | 0.38917 | 0.545931 | 0.344376 |
| 31 | 0.380752 | 0.557306 | 0.295948 | 0.383227 | 0.546367 | 0.342789 |
| 32 | 0.373364 | 0.563837 | 0.29408 | 0.376269 | 0.547834 | 0.347 |
| 33 | 0.366838 | 0.567744 | 0.305228 | 0.371107 | 0.551375 | 0.354988 |
| 34 | 0.359827 | 0.57315 | 0.321135 | 0.366092 | 0.552884 | 0.362179 |
| 35 | 0.35348 | 0.576678 | 0.322745 | 0.36128 | 0.554545 | 0.366135 |
| 36 | 0.345351 | 0.576879 | 0.320879 | 0.356888 | 0.553557 | 0.369745 |
| 37 | 0.337853 | 0.579245 | 0.333505 | 0.35133 | 0.556852 | 0.380187 |
| 38 | 0.330348 | 0.579945 | 0.334055 | 0.34896 | 0.556425 | 0.393312 |
| 39 | 0.325985 | 0.58194 | 0.350666 | 0.345874 | 0.559653 | 0.407833 |
| 40 | 0.314002 | 0.580532 | 0.356735 | 0.343833 | 0.559803 | 0.417901 |
| 41 | 0.311194 | 0.581872 | 0.375023 | 0.341981 | 0.560539 | 0.430216 |
| 42 | 0.304963 | 0.582039 | 0.393133 | 0.34138 | 0.559231 | 0.435087 |
| 43 | 0.30065 | 0.586882 | 0.415467 | 0.34022 | 0.55897 | 0.442265 |
| 44 | 0.301101 | 0.588801 | 0.420139 | 0.341258 | 0.559913 | 0.453062 |
| 45 | 0.301011 | 0.589171 | 0.424421 | 0.342976 | 0.562234 | 0.465563 |
| 46 | 0.298366 | 0.585457 | 0.43187 | 0.347345 | 0.56381 | 0.475276 |
| 47 | 0.302467 | 0.584977 | 0.436272 | 0.350902 | 0.564667 | 0.481914 |
| 48 | 0.306069 | 0.586641 | 0.454356 | 0.356686 | 0.566429 | 0.488601 |
| 49 | 0.317827 | 0.584386 | 0.463327 | 0.362341 | 0.566938 | 0.49439 |
| 50 | 0.329425 | 0.585219 | 0.468228 | 0.370263 | 0.566659 | 0.497246 |

**Supplemental Table 4.** Overall ICCs (averaged across the density of 1% to 50%) of all metrics at the subnetwork level. ATT, attention subnetwork; AUD, auditory subnetwork; CON, cinguloopercular subnetwork; DMN, default-mode subnetwork; FPN, frontoparietal subnetwork; SAL, salience subnetwork; SM, sensorimotor subnetwork; SUB, subcortical subnetwork; VIS, visual subnetwork.

| Subnetwork | AAL atlas | | | Power atlas | | |
| --- | --- | --- | --- | --- | --- | --- |
|  | Overall ICC | | | Overall ICC | | |
|  | Temporal clustering coefficient | Clustering coefficient | Local efficiency | Temporal clustering coefficient | Clustering coefficient | Local efficiency |
| DMN | 0.422344 | 0.404607 | 0.294916 | 0.476202 | 0.458195 | 0.400104 |
| SAL | 0.399478 | 0.405523 | 0.291724 | 0.453517 | 0.381909 | 0.297713 |
| VIS | 0.496451 | 0.514545 | 0.457768 | 0.558315 | 0.518073 | 0.502967 |
| SUB | 0.415867 | 0.268508 | 0.253822 | 0.488287 | 0.284389 | 0.308403 |
| AUD | 0.524384 | 0.425586 | 0.363107 | 0.516029 | 0.426308 | 0.358219 |
| FPN | 0.42121 | 0.362147 | 0.252563 | 0.471273 | 0.452133 | 0.381777 |
| CON | 0.432968 | 0.400532 | 0.304884 | 0.411381 | 0.37934 | 0.296166 |
| SM | 0.444736 | 0.434626 | 0.342821 | 0.545855 | 0.531421 | 0.502976 |
| ATT | 0.433724 | 0.400187 | 0.322734 | 0.491918 | 0.437857 | 0.373 |

**Supplemental Table 5.** Comparisons on the global temporal clustering coefficient between male and female subjects at each density (raw uncorrected *p* values are presented here).

| Density (%) | AAL atlas | | | Power atlas | | |
| --- | --- | --- | --- | --- | --- | --- |
|  | Comparison | *F* | *p* | Comparison | *F* | *p* |
| 1 | Female > male | 8.222 | 0.004 | Female > male | 14.550 | < 0.001 |
| 2 | Female > male | 12.850 | < 0.001 | Female > male | 17.710 | < 0.001 |
| 3 | Female > male | 14.930 | < 0.001 | Female > male | 18.646 | < 0.001 |
| 4 | Female > male | 15.417 | < 0.001 | Female > male | 18.873 | < 0.001 |
| 5 | Female > male | 14.933 | < 0.001 | Female > male | 18.681 | < 0.001 |
| 6 | Female > male | 14.381 | < 0.001 | Female > male | 18.348 | < 0.001 |
| 7 | Female > male | 13.611 | < 0.001 | Female > male | 17.869 | < 0.001 |
| 8 | Female > male | 12.772 | < 0.001 | Female > male | 17.429 | < 0.001 |
| 9 | Female > male | 12.067 | 0.001 | Female > male | 16.983 | < 0.001 |
| 10 | Female > male | 11.479 | 0.001 | Female > male | 16.569 | < 0.001 |
| 11 | Female > male | 11.112 | 0.001 | Female > male | 16.264 | < 0.001 |
| 12 | Female > male | 10.609 | 0.001 | Female > male | 16.002 | < 0.001 |
| 13 | Female > male | 10.336 | 0.001 | Female > male | 15.629 | < 0.001 |
| 14 | Female > male | 10.045 | 0.002 | Female > male | 15.326 | < 0.001 |
| 15 | Female > male | 9.768 | 0.002 | Female > male | 15.053 | < 0.001 |
| 16 | Female > male | 9.550 | 0.002 | Female > male | 14.807 | < 0.001 |
| 17 | Female > male | 9.617 | 0.002 | Female > male | 14.607 | < 0.001 |
| 18 | Female > male | 9.626 | 0.002 | Female > male | 14.425 | < 0.001 |
| 19 | Female > male | 9.535 | 0.002 | Female > male | 14.316 | < 0.001 |
| 20 | Female > male | 9.502 | 0.002 | Female > male | 14.139 | < 0.001 |
| 21 | Female > male | 9.617 | 0.002 | Female > male | 14.137 | < 0.001 |
| 22 | Female > male | 9.736 | 0.002 | Female > male | 14.033 | < 0.001 |
| 23 | Female > male | 9.834 | 0.002 | Female > male | 14.036 | < 0.001 |
| 24 | Female > male | 9.913 | 0.002 | Female > male | 13.976 | < 0.001 |
| 25 | Female > male | 9.892 | 0.002 | Female > male | 14.028 | < 0.001 |
| 26 | Female > male | 9.941 | 0.002 | Female > male | 13.964 | < 0.001 |
| 27 | Female > male | 9.940 | 0.002 | Female > male | 14.033 | < 0.001 |
| 28 | Female > male | 9.909 | 0.002 | Female > male | 13.845 | < 0.001 |
| 29 | Female > male | 9.720 | 0.002 | Female > male | 13.743 | < 0.001 |
| 30 | Female > male | 9.730 | 0.002 | Female > male | 13.799 | < 0.001 |
| 31 | Female > male | 9.626 | 0.002 | Female > male | 13.848 | < 0.001 |
| 32 | Female > male | 9.432 | 0.002 | Female > male | 13.677 | < 0.001 |
| 33 | Female > male | 9.267 | 0.003 | Female > male | 13.463 | < 0.001 |
| 34 | Female > male | 9.304 | 0.002 | Female > male | 13.251 | < 0.001 |
| 35 | Female > male | 9.061 | 0.003 | Female > male | 12.981 | < 0.001 |
| 36 | Female > male | 8.662 | 0.003 | Female > male | 12.748 | < 0.001 |
| 37 | Female > male | 8.301 | 0.004 | Female > male | 12.489 | < 0.001 |
| 38 | Female > male | 8.136 | 0.005 | Female > male | 12.071 | 0.001 |
| 39 | Female > male | 7.587 | 0.006 | Female > male | 11.704 | 0.001 |
| 40 | Female > male | 7.168 | 0.008 | Female > male | 11.105 | 0.001 |
| 41 | Female > male | 6.824 | 0.009 | Female > male | 10.450 | 0.001 |
| 42 | Female > male | 6.289 | 0.013 | Female > male | 9.899 | 0.002 |
| 43 | Female > male | 5.556 | 0.019 | Female > male | 9.096 | 0.003 |
| 44 | Female > male | 5.007 | 0.026 | Female > male | 8.319 | 0.004 |
| 45 | Female > male | 4.120 | 0.043 | Female > male | 7.526 | 0.006 |
| 46 | Female > male | 3.342 | 0.068 | Female > male | 6.827 | 0.009 |
| 47 | Female > male | 2.659 | 0.104 | Female > male | 6.024 | 0.015 |
| 48 | Female > male | 2.123 | 0.146 | Female > male | 5.279 | 0.022 |
| 49 | Female > male | 1.490 | 0.223 | Female > male | 4.540 | 0.034 |
| 50 | Female > male | 1.055 | 0.305 | Female > male | 3.812 | 0.052 |

**Supplemental Table 6.** Correlations between age and temporal clustering coefficients of the subcortical subnetwork calculated with different window widths (the sliding step length was set at 8 TRs here).

| Window width | Correlations | |
| --- | --- | --- |
|  | AAL atlas | Power atlas |
| 56 TRs | Spearman’s rho = 0.186,  corrected *p* = 0.005 | Spearman’s rho = 0.191,  corrected *p* = 0.003 |
| 83 TRs | Spearman’s rho = 0.185,  corrected *p* = 0.006 | Spearman’s rho = 0.188,  corrected *p* = 0.005 |
| 111 TRs | Spearman’s rho = 0.178,  corrected *p* = 0.009 | Spearman’s rho = 0.184,  corrected *p* = 0.006 |
| 139 TRs | Spearman’s rho = 0.174,  corrected *p* = 0.012 | Spearman’s rho = 0.173,  corrected *p* = 0.013 |
| 208 TRs | Spearman’s rho = 0.172,  corrected *p* = 0.013 | Spearman’s rho = 0.165,  corrected *p* = 0.021 |

**Supplemental Table 7.** Correlations between age and temporal clustering coefficients of the subcortical subnetwork calculated with different sliding step lengths (the window width was set at 139 TRs here).

| Sliding step length | Correlations | |
| --- | --- | --- |
|  | AAL | Power |
| 6 TRs | Spearman’s rho = 0.181,  corrected *p* = 0.008 | Spearman’s rho = 0.179,  corrected *p* = 0.009 |
| 8 TRs | Spearman’s rho = 0.174,  corrected *p* = 0.012 | Spearman’s rho = 0.173,  corrected *p* = 0.013 |
| 14 TRs | Spearman’s rho = 0.170,  corrected *p* = 0.016 | Spearman’s rho = 0.175,  corrected *p* = 0.011 |
| 28 TRs | Spearman’s rho = 0.154,  corrected *p* = 0.041 | Spearman’s rho = 0.163,  corrected *p* = 0.023 |
